# Factors of the policy process influencing Health in All Policies in local government: A scoping review

**DOI:** 10.3389/fpubh.2023.1010335

**Published:** 2023-02-09

**Authors:** Kara Lilly, Bridie Kean, Jonathan Hallett, Suzanne Robinson, Linda A. Selvey

**Affiliations:** ^1^School of Health, University of the Sunshine Coast, Maroochydore, QLD, Australia; ^2^School of Population Health, Curtin University, Perth, WA, Australia; ^3^Deakin Health Economics, Faculty of Health Sciences, Deakin University, Burwood, VIC, Australia; ^4^School of Public Health, Faculty of Medicine, The University of Queensland, Brisbane, QLD, Australia

**Keywords:** healthy public policy, municipality, political science, policy theory, determinants of health, health equity, health promotion

## Abstract

**Objectives:**

This review aimed to identify factors in the policymaking environment that influence a Health in all Policies approach in local government, how these vary across different municipal contexts, and the extent that theories of the policy process are applied.

**Methods:**

A scoping review was conducted to include sources published in English, between 2001 and 2021 in three databases, and assessed for inclusion by two blind reviewers.

**Results:**

Sixty-four sources were included. Sixteen factors of the policy process were identified, expanding on previously reported literature to include understanding and framing of health, use of evidence, policy priority, and influence of political ideology. Eleven sources applied or referred to theories of the policy process and few reported findings based on different local government contexts.

**Conclusion:**

There are a range of factors influencing a Health in All Policies approach in local government, although a limited understanding of how these differ across contexts. A theory-informed lens contributed to identifying a breadth of factors, although lack of explicit application of theories of the policy process in studies makes it difficult to ascertain meaningful synthesis of the interconnectedness of these factors.

## Introduction

Improving the health of populations requires a shift from individual behavior approaches and advances in healthcare to addressing social, environmental, political, economic and physical determinants of health (1). Influencing health determinants such as housing, transport, sustainability, social services and infrastructure design requires cooperation across all sectors, including those outside of the healthcare system. This approach is referred to as Health in All Policies (HiAP) (2).

Local government (LG), also referred to as municipalities, operate across a diverse range of sectors with the responsibility for urban planning of neighborhoods, transport options, employment conditions, establishment of accessible local facilities and contribution to the social capital of communities (3). While there are different governance structures and legislative environments of LG between countries, LG are universally the closest tier of government to the community, able to engage and connect with the public and create collaborative opportunities between different sectors (3). For these reasons, LG are deemed the most feasible tier of government to address health determinants across a range of policy areas (4–6). However, there is little attention in the literature given to how LG policymaking environments make these decisions to address health determinants (or not).

To date, there have been two scoping reviews that have identified themes that enable or challenge LG in implementing a HiAP approach, locating literature up to 2015 (7) and 2016 (8). Findings from these reviews identify key enablers such as available funding and strong leadership or champions in both local and higher tiers of government (7, 8), along with community engagement (7). The role of national legislation is also considered a facilitative tool in the policy process, particularly if allowing for autonomy at a local level (7, 8). Health impact assessments are acknowledged as useful, albeit require political commitment, training and support to be feasible (8). The importance of intersectoral collaboration is an identified facilitator for achieving implementation of HiAP, although it was deemed an extra task for sectors outside of health, and requires skills in effective communication (7, 8). In addition, barriers to achieving a HiAP approach include lack of funding, lack of clear objectives and performance indicators to measure health outcomes (7, 8), lack of ownership and accountability for HiAP at a local level (8), lack of staff expertise, and siloed organizational structures (7).

Whilst the themes identified across these two reviews identify a range of distinctive enablers and challenges to the policy process, more recent health promotion discourse proposes that understanding decision-making processes could be better understood by exploring the policymaking environment through application of theories of the policy process (9, 10). By understanding the factors that influence the policymaking environment, practitioners could better navigate policy decision-making, regardless of the unique LG contexts, whether that be within or between countries, rural and city locations or legislative arrangements (10, 11). However, it seems that whilst health promotion policy research is increasing, few are informed by theories of the policy process, or continue to rely on simplified and outdated stages heuristic models (10, 12, 13). The stages heuristic model, whilst easy to understand for health promotion practitioners, does not reflect the messiness and interrelatedness of the complexity of factors influencing the policy process (9, 14). Theories of the policy process, however, give meaning to the interaction of many factors, such as political environments, policy actor beliefs and interests, public opinions, events, political ideologies and power to name a few (15, 16).

Informed by theories of the policy process, this scoping review identifies a more in-depth perspective of the policy process, by capturing the most recently available research on factors that influence the policy process (inclusive of enablers and challenges), including an exploration of how the research gives consideration to different legislative and geographical LG contexts. Alongside this, the review determines the extent that current research explicitly applies theories of the policy process when exploring the role of HiAP in the LG policymaking environment. It extends upon previous findings to identify gaps in the literature and provide discussion on future directions for research in HiAP in the LG policymaking environment.

HiAP remains an emerging topic and advancing our knowledge could be enhanced by incorporation of evidence from both empirical and gray literature (17). Therefore a scoping review was deemed an appropriate approach for advancing the field. Scoping reviews are valuable in health as the method collates, organizes and interprets large volumes of evidence on a specific topic (18) and the exploratory nature of the research questions aligns with the value of scoping reviews in synthesizing and mapping concepts, types of evidence, and research gaps (18).

The research question proposed for this review was:

“What are the factors in the policy process that enable and/or challenge LG in initiating, implementing or evaluating a HiAP approach to achieve population health and wellbeing outcomes?”

Additional sub-questions of interest included:

- How does the literature related to a HiAP approach in LG apply theories of the policy process?
- Are policy factors related to a HiAP approach different across various LG contexts and jurisdictions?

## Methods

The scoping review method was conducted in accordance with Joanna Briggs Institute (JBI) methodology for scoping reviews (19) and recommendations for conducting scoping reviews (18). A full outline of the protocol is provided open access (20). The research question guided the process to identify all relevant literature (18). The initial step included a search of relevant literature from 2001 to January 2021, reflecting 20 years of health promotion policy research, across Scopus, Proquest and EBSCO. The databases were selected for their multidisciplinary focus across health, politics and humanities, with Proquest including gray literature sources. Included terms were considered based on the goal of obtaining sources of evidence that address population health and wellbeing, rather than those with a focus on health care, health services, or individual approaches to health and wellbeing. The research team have content expertise required to guide the review (18), and in this case it assisted in determining appropriate terminology to include in the search strategy. Based on knowledge and extensive experience in health promotion by several authors (KL, JH, LS), and HiAP being a relatively new term in health promotion, it was deemed necessary to include both the concept “HiAP” in the search terms, as well as additional relevant concepts, such as structural determinants of health [political, social, environmental (built or natural)] or concepts such as health equity. The search strings are outlined in Table 1.

**Table 1:** Database search, including search strings, limits and number of sources (after removing duplicates within databases).

**Database**	**Search string used**	**Limits**	**Number of sources**
Proquest	(“local government” OR “municipality” OR “local council” OR “city government”) AND (“health in all polic^*^” OR “determinants of health” OR “health inequit^*^” OR “health equit^*^”) AND (“policy process”)	**Year:** 1 Jan 2001–1 Jan 2021 **Language:** English **Sources:** Dissertations and Theses, Scholarly Journals and Reports	593
Scopus	(“local government” OR “municipality” OR “local council” OR “city government”) AND (“health in all polic^*^” OR “determinants of health” OR “health ^*^equit^*^”) AND (“polic^*^“)	**Year:** 2001–12 March 2021 **Language:** English **Sources:** Articles and Reviews	208
EBSCO	(“local government” OR “municipality” OR”local council” OR “city government”) AND (“health in all polic^*^” OR “determinants of health” OR “health ^*^equit^*^”) AND (“polic^*^”)	**Year:** 2001–Jan 2021 **Language**: English **Sources:** Academic Journals, Journals, Dissertations	234

Sources needed to address any factor related to the policy process, be positioned in the LG context and address HiAP or a related concept. A full list of inclusion and exclusion criteria are summarized in Table 2.

**Table 2:** Summary of inclusion and exclusion criteria applied to the sourcing of evidence.

	**Inclusion criteria**	**Exclusion criteria**
Context	LG context; Multiple tiers of government involved, although only if the role of LG was clear.	Set in state or federal tiers of government. Set in local healthcare settings or community-based organizations. Not in any particular context.
Concept 1	Address HiAP or a related concept such as determinants of health, health equity.	Address health with reference to biomedical or healthcare approach or a focus on individual health (including urban planning environment and built environment related to individual health risk factors).
Concept 2	Discuss any factor related to the policy process.	Discuss policy content or policy impacts/outcomes.

An initial list of possible factors influencing the policy process were available to all reviewers to assist in the assessment of articles for inclusion, particularly as one reviewer was less familiar with the theories of the policy process. However, the reviewers were not bound to only these initial factors. Any sources that presented research of factors influencing the policy process were discussed and included if they otherwise met the criteria. The initial factors were informed by a deconstruction of concepts related to theories of the policy process, in specific reference to the Multiple Streams Framework (MSF) (21), Advocacy Coalition Framework (ACF) (22), Punctuated Equilibrium Framework (PEF) (23) and Analysis of Determinant of Policy Impact (ADEPT) (24). The first three frameworks were chosen to inform the inclusion criteria as they are considered three of the most established and rigorously tested frameworks in the policy sciences, albeit largely focussed on policy agenda setting (15). The ADEPT framework, initiated from and adapted from health promotion behavior change models, aimed to address the gaps in factors related to policy implementation and evaluation (25). A mind map of the policy process concepts deconstructed from these theories is available in the scoping review protocol (e.g., is availabe in the scoping review protocol (see Supplementary material).

Articles obtained in the search were assessed for inclusion by two blind reviewers (KL, BK) at title/abstract level using Rayyan software (26). If it was not clear whether the article referred to population or individual health and wellbeing, LG or other tiers of government or a HiAP related concept the article was included at full-text review. The reference lists of sources that met inclusion criteria were scanned to identify any additional sources. Two authors [initials removed for peer review] reviewed all articles at full text and discussed any discrepancies during regular reviewer meetings (18). In one instance where the context remained unclear, the primary author was contacted to clarify. All evidence sources were included that met the criteria, excluding reviews of the literature.

Data from articles included at full text review were extracted for further analysis. Data extraction included key concept, nation of article, use of theory and key findings of the study (see Supplementary material). Data extraction also included commentary on how findings were compared across different LG contexts, for example size of LG or different legislative environments. Two authors conducted this process for approximately 10% of the included articles, checking the data extracted for consistency (18). The first author completed this process for the remaining articles included at full-text review.

The final stage of the scoping review involved an inductive, qualitative thematic analysis of the key findings, completed by two authors (KL, BK). The first author (KL) conducted a first pass of the data manually and developed initial codes through an inductive approach. Using these initial codes as a guide, two of the authors coded from all datasets. Following blind data analysis, the two authors discussed the coded dataset and continued the qualitative data analysis using NVivo 12 software [QSR International Pty Ltd. (2018) NVivo (Version 12), https://www.qsrinternational.com/nvivo-qualitative-data-analysis-software/home] to build a consensus of themes that represented key factors of the policy process (27). Through this process, factors of the policy process and their relationships to one another were critically discussed. Other aspects of the literature critically discussed in depth during this process were: (1) the use of theory to inform study design and interpretation of findings across the body of literature, (2) inconsistencies with terminology, and (3) how to capture the interconnectedness of themes.

## Results

**Figure 1:**
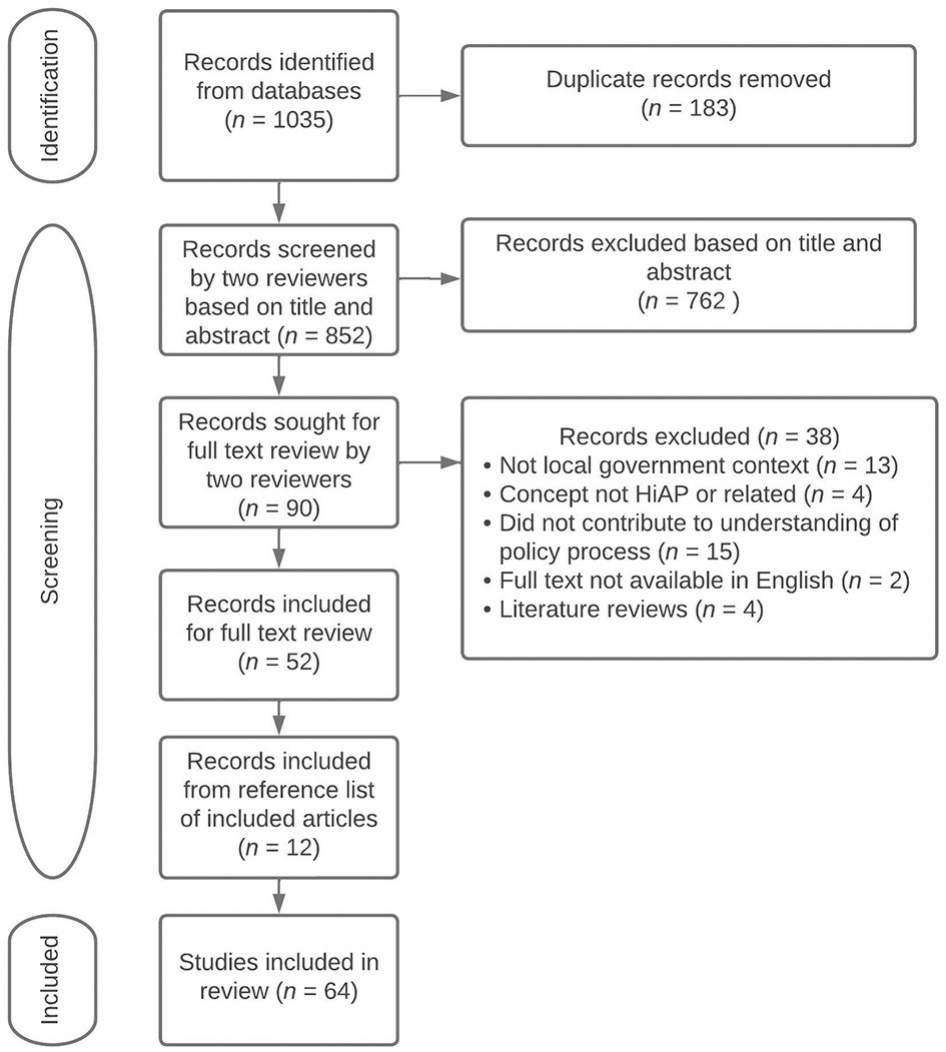
PRISMA flow chart detailing the number of sources included at each stage of the review process, including reasons for exclusion at full text.

The initial database search yielded 1,035 sources for possible inclusion in the review. After removing duplicates, 852 sources were assessed for inclusion based on title and abstract, resulting in 90 sources read in full text. Of these, 52 sources were deemed to meet the inclusion criteria. A scan of the references of sources that met the inclusion criteria identified another 12 sources eligible for the review. A total of 64 sources were included in the final review (Figure 1).

**Figure 2:**
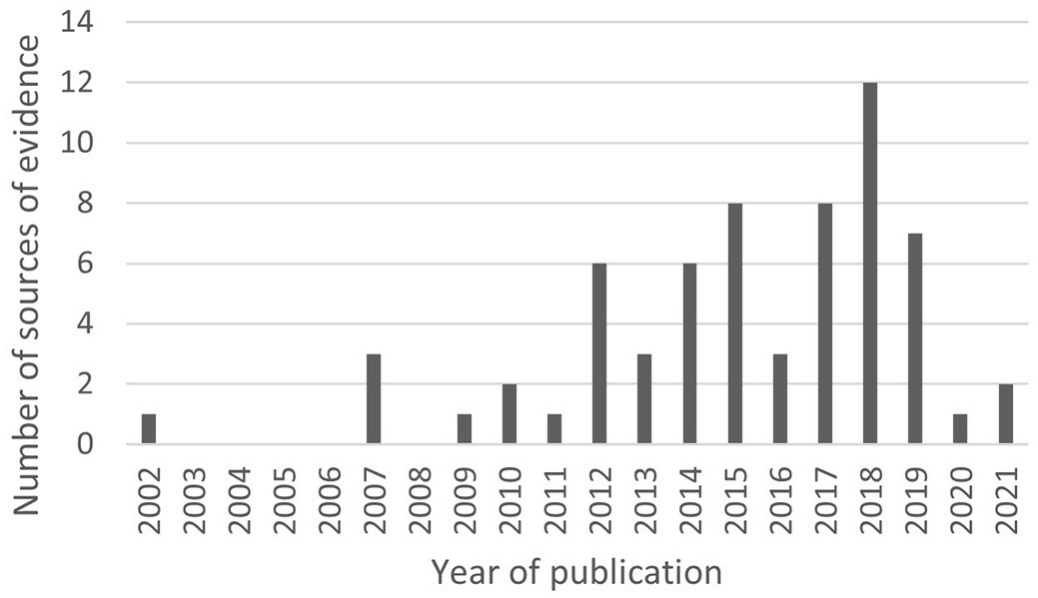
Year of publication of included sources.

Of the included sources, 56 (87%) were published since 2012, and 30 (47%) since 2017 (Figure 2).

Sources represented a range of countries, including Norway (15, 23%), followed by Australia (10, 15%), Netherlands (8, 12.5%), Denmark (6, 9%), England (6, 9%), Sweden (4, 6%) and United States of America (4, 6%). Other countries less represented in the literature were the United Kingdom (3, 5%), Canada (3, 5%), Brazil (2, 3%) and single studies from Cuba, Finland, Hungary, Slovakia, Spain, Turkey, Europe and one not an empirical study. A majority were exclusively undertaken within the LG context (59, 92%) and did not address other tiers of government. Twenty-one sources of evidence were focussed on HiAP as a concept (33%), with others exploring relevant concepts such as determinants of health (17, 27%), health equity (13, 20%), or less defined concepts such as health promotion, public health or healthy planning (13, 20%).

### Factors identified in the policy process

Amongst the included sources, there were 16 factors identified as influencing the policy process (Table 3). Some sources focussed on a single factor (e.g., intersectoral collaboration), whilst others included a range of factors. The 16 identified factors influencing the policy process are summarized in Table 3 and the next section reports in more detail on the novel findings of the review, including any insight into contextual variances of LG based on size, geographical location or legislative environment.

**Table 3:** Outline of the 16 identified factors influencing HiAP within LG, with a summary of the key findings (review, global, 2011–2021).

**Theme**	**Frequency (*n*, %)**	**Key findings**	**References**
Cross-sector relationships	44, 69%	Collaboration across sectors was regularly reported as necessary for HiAP, though challenging to achieve. There was more focus on horizontal collaboration across departments, than vertical collaboration between staff and decision-makers.	(5, 28–70)
Evidence	34, 53%	LG utilizes a wide range of evidence sources. Local data, including community input, was consistently reported as more important than academic sources of evidence.	(30, 32–37, 40, 42–44, 47, 49–52, 55, 57–60, 66, 67, 71–81)
Level of policy priority	26, 40%	Addressing health determinants was reported as a priority for LG, though not always the highest priority, amongst more politically favorable lifestyle programs and other competing LG interests.	(5, 29, 30, 36, 38–48, 51, 53, 54, 59, 64, 68, 70–72, 82, 83)
Understanding of health	24, 38%	The definition and understanding of the term “health” was perceived as ambiguous and complex, and varied amongst decision-makers.	(5, 28–32, 35, 40, 42–44, 46, 51, 60, 62, 65, 68, 69, 71, 72, 82–85)
Funding	23, 36%	Many sources highlighted the challenge of financial constraints, or reliance on higher tiers of government for funding.	(5, 29, 30, 32, 35, 37, 40, 42, 46, 48, 49, 51, 58, 59, 61, 63, 64, 68, 70–72, 75, 82)
Leadership/Political commitment	18, 30%	Support from local management and politicians was reported as a key contributor to local policy success.	(29, 32, 34–36, 38, 39, 46, 49, 53, 59, 60, 62, 64–66, 68, 86)
Champions and policy entrepreneurs	16, 25%	Champions were reported as important in initiating HiAP, although not necessarily existing in LG.	(29, 30, 34–36, 39, 42, 45, 52–54, 58, 63, 71, 81, 86)
Framing	15, 23%	LG decision-makers often referred to “health” as another concept such as liveability, or wellbeing. Rarely was “health” the reason for action on addressing determinants of health.	(28, 30–39, 53, 57, 65, 84)
Role of community	14, 22%	Community input is a key influence in local level policy decision-making. There is some debate over the level of comfort by planners in trust that community will focus on determinants of health, if engaged in the process.	(28, 32, 34, 36, 37, 40, 46, 49, 51, 54, 59, 60, 82, 87)
Role of legislation	14, 22%	Legislation was reported as a contributor to successful initiation and implementation of HiAP, although LG did not always adhere to the mandate, and required sufficient resourcing.	(35, 36, 44–49, 52, 60, 69, 71, 75, 82)
Staff capacity	12, 19%	Capacity of staff time and expertise was reported as a challenge to a HiAP approach.	(29, 32, 36, 46, 49, 53, 59, 61, 67, 73, 78, 88)
Use of tools (e.g., Health impact assessment)	11, 17%	Health impact assessments were reported as useful to assessing possible health impacts across sectors and raising awareness of health determinants amongst policy decision-makers, although challenged by lack of legislation and adequate resourcing.	(33, 43, 48, 54, 57, 61, 62, 66, 69, 71, 89)
Political ideology and decision making	10, 16%	Broader political ideologies, and individual values and beliefs, influenced the commitment to addressing health equity at a local level.	(29, 32, 34, 36, 48, 49, 69, 72, 73, 80)
Responsibility of local government	9, 14%	Health inequalities is accepted as a responsibility of LG, although there is a perceived lack of power or authority to take action.	(5, 39, 40, 43, 44, 51, 53, 69, 72)
Performance measures	9, 14%	Several sources note the lack of, or use of vague performance indicators, contributing to a lack of urgency to address health inequities.	(33, 35, 43, 45, 49, 55, 59, 60, 90)
Organizational structures	5, 8%	There is ongoing debate on a successful governance structure for HiAP in LG, between a centralized unit and cross-department collaborations.	(28, 37, 41, 53, 55)

#### Understanding and framing of health

It is evident from the findings that there is not a unanimous understanding of HiAP or related concepts such as health equity within a LG context. HiAP is reported as complex and difficult to define (84). In terms of understanding, there was evidence of LG staff knowing the impact of policies on health (5, 28, 29, 82), as well as examples of LG staff and decision-makers conversely regarding health as the responsibility of individuals (30–32, 83).

The broader terminology of HiAP (inclusive of social determinants of health and health inequities) was perceived to gain little policy attention in LG (28, 30–34). Whilst LG appeared to address health determinants, some of the findings suggested health was not the prominent reason for action that led to better health outcomes (31, 35–39). Alternative framing of the issue, namely as liveability, wellbeing (36) or living conditions (31, 84) was more accepted, although recognized as shifting the focus away from health (31, 33). Synnevåg et al. (84) suggest the term “public health” should be used to gain support, though concede it may need to be reframed during the process to be more relevant to different contexts.

#### Level of policy priority

Health inequalities or health determinants were consistently regarded as a high policy priority (30, 40, 82), despite competing with other LG responsibilities (29, 41). Some studies reported that only a select few LGs regarded health determinants a high priority (5, 42). Behavioral and lifestyle programs were perceived to be of higher priority in LG compared to addressing determinants of health (43, 44, 71, 83). The level of policy priority for addressing determinants of health was challenged by a range of factors including the political attraction to lifestyle programs (82), the perceived lack of urgency (45) and competing with priorities such as healthcare, food safety or other nationally determined priorities (44, 46, 72, 83). Larger municipalities in Norway were more likely to prioritize policies regarding living conditions, while smaller municipalities prioritized lifestyle issues (44, 47).

#### Political ideology and decision making

Politicized decision-making was also a challenge faced in the LG context, recognizing that population health outcomes extend beyond short term political election timeframes (36, 72) and misalign with political ideologies for those seeking economic growth (48). Commitment to health inequities was higher if it aligned to the values and ideologies of decision-makers (34). Commitment was lower where values conflicted or where political reputations were seen to be at risk (48, 49, 73).

#### Evidence

The review found that local data, such as anecdotal experiences and knowledge, is considered more important than academic sources of evidence in the LG context (49, 72, 74), although accessing relevant local data is acknowledged as difficult (36, 49, 50, 75–77). Browne et al. (76) found that academic research was the least utilized evidence input was the most commonly used and influential type of evidence (49, 72, 73, 76, 77), along with anecdote (73, 74), networks (30, 76, 77), media (30, 77) or government reports (30, 36, 51, 76, 77).

Evidence was more likely to support policy action if it highlighted health inequities (34, 40), included a cost effectiveness argument (33, 73, 78), was free of jargon (73), was already synthesized (78), was politically acceptable and viable (74, 78), and appealed to values of doing the “right thing” (78). However, some authors caution that even where local level data is available, it may not be used to inform policy (52). Sources reported some key challenges for LG utilizing evidence, such as the limited time of staff (76–79) and availability of evidence (77, 79).

#### Champions and policy entrepreneurs

Findings from this scoping review confirms the importance of the role of a policy entrepreneur, or individual in a similar role such as a champion or knowledge broker (35, 45, 53, 86). However, whilst the potential value of a policy entrepreneur was consistently identified, few studies reported on either the presence of, or role of such entrepreneurs in the LG context (29, 54).

#### Organizational structures

The review unveiled a debate on the influence of organizational structures that support collaboration across sectors. There was no conclusive agreement on the ideal governance structure. Holt et al. (41) report that a central unit was difficult for public health staff to engage other sectors, whilst Von Heimburg et al. (55) found the contrary. Other organizational structures that support formal communication across departments are proposed as useful, such as intersectoral committees (56, 57), or formal structures such as strategic planning and health impact assessments (57). Additional findings suggest that beyond formal communication and structures, intersectoral collaboration requires a range of interpersonal skills to be effective, such as the development of trust between sectors (53, 55), conflict management (37) and communication skills (28).

#### Funding

The literature highlighted the challenge of financial constraints (29, 32, 40, 51, 58, 59). However, whilst agreeing funding is an important component, it is debatable whether the size of LG has an influence on the level of resources and capacity to address health inequities. Bekken et al. (75) report that larger Norwegian municipalities are more likely to be sufficiently resourced compared to smaller municipalities, which is in alignment with results by Lilly et al. (29) that city councils were better resourced than regional and rural counterparts. However, Bagley et al. (46) found no difference in the approach to municipal public health planning between Australian LGs in relation to their level of wealth.

### Use of theory in literature

A sub-question in the review was to explore the application of theories of the policy process in the literature. A third (*n* = 23) of the included sources utilized any type of theory, varying between social, organizational or political science theories (see Supplementary material). There were 11 sources that applied or referred to theories of the policy process. Of these, nine applied the MSF, or referenced an aspect of the framework relevant to the research. For example, Hoeijmakers et al. (54) applied only the policy entrepreneur component of the MSF. Five sources applied the MSF explicitly to inform or interpret the research findings (29, 36, 45, 66, 86), while others mention the MSF, but do not discuss how it informed the study design, results or interpretation of findings (33, 35, 55). Other frameworks that were applied included network analysis (54), or other conceptual frameworks adapted for exploration of the policy process (34, 44). For example, Fosse et al. (44) applied the Gradient Evaluation Framework, which had been adapted from the stages heuristic policy framework to evaluate the extent to which policies considered health inequities. The use of multiple theories was applied by three sources (29, 35, 54).

## Discussion

This review served two purposes: (1) identify the factors in the policy process that enable and/or challenge a HiAP approach in LG, including across different jurisdictions and (2) explore how literature exploring HiAP approaches in LG applied theories of the policy process.

### Factors in the policy process

The recent literature, up to 2021, confirms many findings previously reported in scoping reviews up to 2016, including influencing factors such as funding, leadership, intersectoral collaboration, clear objectives and performance indicators (7, 8). The wide range of factors demonstrate the complexity of the policy process. Some factors were clearly enabling the process, such as the level of priority given to health and wellbeing and strong leadership within LG. Key challenges in the LG policymaking environment were lack of key champions, limited funding and staff capacity, difficulty collaborating across sectors, the ambiguous responsibility of LG to respond to HiAP, and the lack of performance measures for health and wellbeing indicators. Finally, there were many factors that were recognized as influential, however, neither a clear enabler nor challenge to the policy process. This is consistent with findings from other authors who suggest that factors of the policy process influencing social determinants of health inequities instead be referred to as “increasing (or decreasing) the “probability”” of influencing the policy process (91) [p. 108].

This scoping review added new themes not previously captured in published reviews on this topic, such as framing, level of policy priority, the role of evidence and political ideologies. Additional insights were identified for the role of policy entrepreneurs or champions, organizational structures and possible contextual considerations regarding funding. The theoretical lens adopted in this scoping review may have contributed to the identification of additional themes in the policy process, not previously reported. Also, it is likely the additional themes were a result of the emerging literature exploring HiAP in LG. For example, the theme related to use of evidence was predominantly identified in literature post 2015. None of the themes identified in this review contradict previous findings, rather extend upon our understanding that the role of HiAP in the LG policy environment is multifactorial, supporting the call for the application of theories of the policy process to understand the complexity and scope of factors related to the policy process.

### Use of theory

This review explored how theories of the policy process were applied in the literature, finding that most of the sources did not apply any type of theory. Whilst the lack of theory did not prohibit the sources from identifying a wide scope of factors in the policy process being identified, the use of theory can provide a way to understand the complexity of how factors are interconnected.

The scoping review identified only 11 sources that applied theories of the policy process, but very few that had applied the theories explicitly and comprehensively in the communication of the research methodology and findings. Given the MSF was the most commonly applied framework in the review studies, an attempt was made to synthesize the findings to demonstrate how this framework could explain the interconnectedness of policy factors. The MSF claims that three streams must align for policy action to be realized (21). To understand this in the policymaking environment requires exploration of how policy issues were raised on the LG policy agenda, referring to factors such as the role of evidence, framing or policy actor involvement (problem stream), and then in regard to how these same factors influence action on health determinants (policy stream) and under what type of political, legislative and governance contexts (politics). For example, whilst the term health was not explicitly discussed amongst LG decision-makers, there is not the broader understanding of whether this impacts on policy solutions to address health determinants, or what other influences within the political environment enable or constrict these decisions from reaching the policy agenda given “health” *per se* may never be discussed. Most of the studies did not comprehensively assess the policymaking environment to make the connections across the three streams of the MSF. In fact, this was the case regardless of whether the framework was applied or not. Despite attempts, a comprehensive synthesis was not achievable given the limited number of studies, the different concepts that were being studied (healthy planning, health inequalities, health impact assessment and health determinants) and across three different countries, with varied governance structures. Even where findings could potentially be synthesized, it was apparent the MSF was applied in slightly different ways or raised different considerations within each of the theoretical streams. For example, the cooperation across sectors and LG departments was often interpreted similarly within the policy stream (29, 36, 45, 66), all agreeing that this was limited and challenging the policy process. In contrast, the influence of higher tiers of government was referred to in either the policy stream (36) or in the politics stream (29). Until there is a sufficient volume of empirical studies that effectively and explicitly apply these theories, it remains difficult to synthesize the interrelatedness of factors that influence the policy process in a meaningful way. Accordingly, it is impractical to propose any evidence-informed suggestions on how to change the practices of health promotion to influence the policy process. This supports both the need for health promotion practitioners and academics to further embrace the political nature of health promotion to inform future practice and collaborate with experts in political science for a more accurate and consistent application of the theories (13, 92). Accordingly, findings from this review support the call for health promotion to apply theories of the policy process to effectively inform practice and research in navigating the policy process. As previously highlighted by experts in health promotion, the globally recognized professional knowledge and skill competencies to achieve this are currently insufficient (9). These professional competencies for health promotion require updating to support international health promotion practitioner registration, along with a contemporary curriculum focussed on politics within related tertiary education degrees (11, 93).

### Contextual differences

The scoping review extended upon previous reviews to explore differences across various LG contexts. However, across the literature very few sources reported findings based on the context within LG e.g., size, rurality or responsibilities. Within the limited existing literature, larger, metropolitan settings appear to have a greater understanding and prioritization of health determinants as a policy issue, and the resource capacity for a HiAP approach.

Furthermore, it is important to note sources were predominantly located in countries where there are legislative environments and existing governance structures to support action on addressing health determinants at a local level. However, often the description of the governance structures was not comprehensive, resulting in difficulty in making comparisons based on geographical locations. Based on this review, it is recommended that future research provides a comprehensive overview of the size, location and any other key descriptors of LG settings to enable findings to be better contextualized. Further research is needed to disaggregate and communicate findings based on these different contexts, rather than combining the findings together as a whole. In terms of geographical spread, the majority of the research was located in European countries, which limits the scoping review from providing a global perspective. More research is required to contribute to this research gap in other countries not represented, particularly countries where there is no legislative requirement for HiAP within LG.

### Limitations

There were several limitations in the conduct of this scoping review. It is acknowledged that the method taken for the scoping literature review meant that some sources that were not intentionally addressing the comprehensiveness of the policy process may have been included. For example, some sources focussed on a single factor such as evidence or health impact assessments. However, across the body of literature, most sources identified an extensive range of factors influencing the policy process, as opposed to narrowing in on one specific factor. However, the sources did not analyse or present factors in a similar or comparative way.

Only studies published in English were included. Given the higher volume of sources from Scandinavian countries, this may have restricted the volume of existing literature on this topic. Given LGs in countries such as Norway have legislative requirements for HiAP, there may be more relevant and indepth studies available in non-English language sources not accessible to the authors of this review. The range of sources, including gray literature, may also have been expanded if more databases were included in the search (e.g., PubMed, Google Scholar).

The search terms were broadened to attempt to incorporate all sources related to HiAP as well as terms that are often used interchangeably with the concept. Whilst the use of different terminology may have broadened the research scope, in some instances the terminology of the key concept used by authors was not clear (e.g., health promotion) and these sources were included. However, our interpretation of these concepts may have differed to the original source intent. This highlights the need to be clearer in the terminology used, as determined more broadly in public health discourse (94). The different governance structures of LG across countries were sometimes difficult to ascertain. However, all action was taken to minimize the exclusion of articles as a result of not knowing the political structures, including contacting primary authors to clarify.

In addition, whilst policy science researchers recognize there is no “ideal” framework (15), the inclusion of sources was initially guided by factors of the policy process as deconstructed from only four frameworks, and as is the case with the MSF and ACF, mostly tested in national tiers of government (95, 96). Therefore, these frameworks may not explain all of the factors of the policy process, or all those relevant to local tiers of government.

Whilst all measures were taken to identify all relevant sources of evidence, there were several sources identified from scanning reference lists, which suggests some other relevant literature may have been inadvertently excluded.

## Conclusion

This scoping review identified a range of factors to the policy process influencing LG adopting a HiAP approach, including newly reported factors such as understanding and framing of health, use of evidence, policy priority, and influence of political ideology. The study concludes that whilst practitioners can learn from the factors that influence the LG policy process to encourage a HiAP approach, it would be more useful to build the evidence-base through the use of theoretical foundations, such as theories of the policy process. Many have previously called for the use of theory to guide the complex policy process, though the actual application in health promotion has been slow in uptake. This scoping review reinforces that this is also the case for studies exploring a HiAP process within the LG setting. Future research exploring HiAP in LG should apply theories of the policy process to be able to comprehensively and meaningfully explain the otherwise messy policy process. A more consistent theoretical application will also allow for further comparison of findings across different LG contexts, including LG size or broader legislative environments.

## Author contributions

KL developed the scoping review protocol, undertook the literature search and was one of the peer reviewers through the review process, led the drafting, writing, and finalization of the manuscript. BK contributed to the finalization of the protocol and methodology for the review, co-wrote the manuscript, contributed feedback and suggestions for clarity, and was a second reviewer in the scoping review process. JH and SR provided input on the peer review process where decisions could not be agreed by KL and BK. SR provided feedback on the initial scoping review methodology. JH read drafts, contributed feedback on the scoping review methodology, and an initial draft of the manuscript. LS contributed by providing feedback on the scoping review protocol, contributing feedback on manuscript drafts, proofreading, and suggestions for amendments for finalizing the manuscript. All authors contributed to the article and approved the submitted version.

## References

[1] Marmot M Friel S Bell R Houweling TA Taylor S. Commission on Social Determinants of Health. Closing the gap in a generation: health equity through action on the social determinants of health. Lancet. (2008) 372:1661–9. 10.1016/S0140-6736(08)61690-6 18994664

[2] World Health Organization and the Government of South Australia. Adelaide Statement on Health in All Policies: Moving Towards a Shared Governance for Health and Well-being. Report from the International Meeting on Health in All Policies; Adelaide, SA: Oxford University Press. (2010). 10.1093/heapro/daq034 20484541

[3] World Health Organization. Addressing the Social Determinants of Health: The Urban Dimension and the Role of Local Government. World Health Organization. Regional Office for Europe. (2012) 56.

[4] Burris S Hancock T Lin V Herzog A. Emerging strategies for healthy urban governance. J Urban Health. (2007) 84:154–63. 10.1007/s11524-007-9174-6 17464568 PMC1891653

[5] Collins PA Hayes MV. Examining the capacities of municipal governments to reduce health inequities: a survey of municipal actors' perceptions in Metro Vancouver. Can J Public Health. (2013) 104:e304–e10. 10.17269/cjph.104.3873 24044470 PMC6974048

[6] Harris E Wills J. Developing healthy local communities at local government level: lessons from the past decade. Aust N Z J Public Health. (1997) 21:403–12. 10.1111/j.1467-842X.1997.tb01722.x 9308206

[7] Van Vliet-Brown CE Shahram S Oelke ND. Health in All Policies utilization by municipal governments: scoping review. Health Promot Int. (2018) 33:713–22. 10.1093/heapro/dax008 28334905

[8] Guglielmin M Muntaner C O'Campo P Shankardass K. A scoping review of the implementation of health in all policies at the local level. Health Policy. (2018) 122:284–92. 10.1016/j.healthpol.2017.12.005 29305241

[9] de Leeuw E Clavier C Breton E. Health policy – why research it and how: health political science. Health Res Policy Syst. (2014) 12:55. 10.1186/1478-4505-12-55 25248956 PMC4246431

[10] Cairney P St Denny E Mitchell H. The future of public health policymaking after COVID-19: A qualitative systematic review of lessons from health in all policies. Open Research Europe. (2021) 1:23. 10.12688/openreseurope.13178.2 PMC10445916 37645203

[11] Greer SL Bekker M de Leeuw E Wismar M Helderman JK Ribeiro S. Policy, politics and public health. Eur J Public Health. (2017) 27:40–3. 10.1093/eurpub/ckx152 29028231

[12] Breton E De Leeuw E. Theories of the policy process in health promotion research: a review. Health Promot Int. (2011) 26:82–90. 10.1093/heapro/daq051 20719803

[13] de Leeuw E Harris P Kim J Yashadhana A. A health political science for health promotion. Glob Health Promot. (2021) 28:17–25. 10.1177/17579759211034418 34510937

[14] Bernier NF Clavier C. Public health policy research: making the case for a political science approach. Health Promot Int. (2011) 26:109–16. 10.1093/heapro/daq079 21296911

[15] Heikkila T Cairney P. Comparison of theories of the policy process. In:Weible C Sabatier P, editors. Theories of the policy process. 4th ed. Boulder, CO: Westview Press. (2018). p. 301–27.

[16] Oneka G Shahidi FV Muntaner C Bayoumi AM Mahabir DF Freiler A. A glossary of terms for understanding political aspects in the implementation of Health in All Policies (HiAP). J Epidemiol Community Health. (2017) 71:835–8. 10.1136/jech-2017-208979 28679539

[17] Levac D Colquhoun H O'Brien KK. Scoping studies: advancing the methodology. Implement Sci. (2010) 5:69. 10.1186/1748-5908-5-69 20854677 PMC2954944

[18] Colquhoun HL Levac D O'Brien KK Straus S Tricco AC Perrier L. Scoping reviews: time for clarity in definition, methods, and reporting. J Clin Epidemiol. (2014) 67:1291–4. 10.1016/j.jclinepi.2014.03.013 25034198

[19] Peters MD Godfrey C McInerney P Munn Z Tricco A Khalil H. Scoping reviews. In:Aromataris E MZ, editor. JBI Manual for Evidence Synthesis. (2020). p. 408–46.

[20] Lilly K Kean B Robinson S Hallett J Selvey LA. Factors of the policy process that influence local government to progress a health in all policies approach: scoping review protocol. Figshare. (2021). 10.6084/m9.figshare.14691414.v1

[21] Kingdon JW. Agendas, Alternatives and Public Policies. 2nd edn. New York, NY: Harper Collins. (1995).

[22] Sabatier P Jenkins-Smith H. The advocacy coalition framework: an assessment. In:Sabatier P, editor. Theories of the Policy Process. Westview Press. (1999). p. 117–66.

[23] Baumgartner F Jones B. Agendas and Instability in American Politics. Chicago, IL: University of Chicago Press. (1993).

[24] Rütten A Gelius P Abu-Omar K. Policy development and implementation in health promotion—from theory to practice: the ADEPT model. Health Promot Int. (2011) 26:322–9. 10.1093/heapro/daq080 21177769

[25] Rütten A Gelius P Abu-Omar K. Action theory and policy analysis: The ADEPT model. In: Health Promotion and the Policy Process. Oxford: Oxford University Press. (2013). p. 174–87.

[26] Ouzzani M Hammady H Fedorowicz Z Elmagarmid A. Rayyan—a web and mobile app for systematic reviews. Syst Rev. (2016) 5:210. 10.1186/s13643-016-0384-4 27919275 PMC5139140

[27] Braun V Clarke V Hayfield N Terry G. Thematic Analysis. Singapore: Springer Singapore. (2019). p. 843–60.

[28] Hendriks AM Jansen MWJ Gubbels JS De Vries NK Molleman G Kremers SPJ. Local government officials[U+05F3] views on intersectoral collaboration within their organization - a qualitative exploration. Health Policy Technol. (2015) 4:47–57. 10.1016/j.hlpt.2014.10.013

[29] Lilly K Hallett J Robinson S Selvey LA. Insights into local health and wellbeing policy process in Australia. Health Promot Int. (2020) 35:925–34. 10.1093/heapro/daz082 31504496

[30] Lawless A Lane A Lewis FA Baum F Harris P. Social determinants of health and local government: understanding and uptake of ideas in two Australian states. Aust N Z J Public Health. (2017) 41:204–9. 10.1111/1753-6405.12584 27774688

[31] Holt DH Frohlich KL Tjørnhøj-Thomsen T Clavier C. Intersectoriality in Danish municipalities: Corrupting the social determinants of health? Health Promot Int. (2017) 32:881–90. 10.1093/heapro/daw020 27006364

[32] Mundo W Manetta P Fort MP Sauaia A. A qualitative study of health in all policies at the local level. Inquiry. (2019) 56. 10.1177/0046958019874153

[33] Scheele CE Little I Diderichsen F. Governing health equity in Scandinavian municipalities: the inter-sectorial challenge. Scand J Public Health. (2018) 46:57–67. 10.1177/1403494816685538 28077033

[34] Schmidt MP Joosen IMPH Kunst AEP Klazinga NSMDP Stronks KP. Generating political priority to tackle health disparities: a case study in the Dutch City of the hague. Am J Public Health. (2010) 100:S210–5. 10.2105/AJPH.2009.168526 20147684 PMC2837449

[35] Jansson EVG Tillgren PE. Health promotion at local level: a case study of content, organization and development in four Swedish municipalities. BMC Public Health. (2010) 10. 10.1186/1471-2458-10-455 20682052 PMC2923108

[36] McCosker A Matan A Marinova D. Policies, politics, and paradigms: Healthy planning in Australian local government. Sustainability. (2018) 10:1008. 10.3390/su10041008

[37] Phillips G Green J. Working for the public health: Politics, localism and epistemologies of practice. Sociol Health Illn. (2015) 37:491–505. 10.1111/1467-9566.12214 25682916

[38] Steenbakkers M Jansen M Maarse H de Vries N. Challenging Health in All Policies, an action research study in Dutch municipalities. Health Policy. (2012) 105:288–95. 10.1016/j.healthpol.2012.01.010 22405487

[39] Storm I den Hertog F Hans van O Schuit AJ. How to improve collaboration between the public health sector and other policy sectors to reduce health inequalities? - A study in sixteen municipalities in the Netherlands. Int J Equity Health. (2016) 15:97. 10.1186/s12939-016-0384-y 27334297 PMC4918104

[40] Morrison J Pons-Vigués M Bécares L Burström B Gandarillas A Domínguez-Berjón F. Health inequalities in European cities: perceptions and beliefs among local policymakers. BMJ Open. (2014) 4:e004454. 10.1136/bmjopen-2013-004454 24871536 PMC4039864

[41] Holt DH Carey G Rod MH. Time to dismiss the idea of a structural fix within government? An analysis of intersectoral action for health in Danish municipalities. Scand J Public Health. (2018) 46:48–57. 10.1177/1403494818765705 29862907

[42] Fosse E Helgesen MK. How can local governments level the social gradient in health among families with children? The case of Norway. Int J Child Youth Family Stud. (2015) 6:328–46. 10.18357/ijcyfs.62201513505

[43] Dhesi SK. Exploring How Health and Wellbeing Boards Are Tackling Health Inequalities with Particular Reference to the Role of Environmental Health (Ph.D.). The University of Manchester, Ann Arbor, United Kingdom. (2014).

[44] Fosse E Sherriff N Helgesen M. Leveling the social gradient in health at the local level: applying the gradient equity lens to norwegian local public health policy. Int J Health Serv. (2019) 49:538–54. 10.1177/0020731419842518 31014169

[45] Exworthy M Berney L Powell M. 'How great expectations in Westminster may be dashed locally': the local implementation of national policy on health inequalities. Policy and Polit. (2002) 30:79–96. 10.1332/0305573022501584

[46] Bagley P Lin V Keating T Wise M Sainsbury P. In what ways does the mandatory nature of Victoria's municipal public health planning framework impact on the planning process and outcomes? Aust New Zealand Health Policy. (2007) 4. 10.1186/1743-8462-4-4 17376248 PMC1851012

[47] Hagen S Torp S Helgesen M Fosse E. Promoting health by addressing living conditions in Norwegian municipalities. Health Promot Int. (2017) 32:977–87. 10.1093/heapro/daw052 27402789

[48] Kokkinen L Muntaner C O'Campo P Freiler A Oneka G Shankardass K. Implementation of Health 2015 public health program in Finland: a welfare state in transition. Health Promot Int. (2019) 34:258–68. 10.1093/heapro/dax081 29149295

[49] Corburn J Curl S Arredondo G Malagon J. Health in all Urban policy: city services through the prism of health. J Urban Health. (2014) 91:623–36. 10.1007/s11524-014-9886-3 25047156 PMC4134455

[50] van der Graaf P Cheetham M Redgate S Humble C Adamson A. Co-production in local government: process, codification and capacity building of new knowledge in collective reflection spaces. Workshops findings from a UK mixed methods study. Health Res Policy Syst. (2021) 19:1–13. 10.1186/s12961-021-00677-2 33514382 PMC7844986

[51] Morrison J Pons-Vigués M Díez E Pasarin MI Salas-Nicás S Borrell C. Perceptions and beliefs of public policymakers in a Southern European city. Int J Equity Health. (2015) 14:18. 10.1186/s12939-015-0143-5 25890326 PMC4343064

[52] Fosse E Helgesen MK Hagen S Torp S. Addressing the social determinants of health at the local level: Opportunities and challenges. Scand J Public Health. (2018) 46:47–52. 10.1177/1403494817743896 29552960

[53] Langeveld K Stronks K Harting J. Use of a knowledge broker to establish healthy public policies in a city district: a developmental evaluation. BMC Public Health. (2016) 16:271. 10.1186/s12889-016-2832-4 26979063 PMC4793512

[54] Hoeijmakers M De Leeuw E Kenis P De Vries NK. Local health policy development processes in the Netherlands: an expanded toolbox for health promotion. Health Promot Int. (2007) 22:112–21. 10.1093/heapro/dam009 17460019

[55] Von Heimburg D Hakkebo B. Health and equity in all policies in local government: processes and outcomes in two Norwegian municipalities. Scand J Public Health. (2017) 45:68–76. 10.1177/1403494817705804 28856984

[56] Spiegel J Alegret M Clair V Pagliccia N Martinez B Bonet M. Intersectoral action for health at a municipal level in Cuba. Int J Public Health. (2012) 57:15–23. 10.1007/s00038-011-0279-z 21845406 PMC3282006

[57] Synnevåg ES Amdam R Fosse E. Intersectoral planning for public health: dilemmas and challenges. Int J Health Policy Manag. (2018) 7:982–92. 10.15171/ijhpm.2018.59 30624872 PMC6326631

[58] Helgesen MK Fosse E Hagen S. Capacity to reduce inequities in health in Norwegian municipalities. Scand J Public Health. (2017) 45:77–82. 10.1177/1403494817709412 28850013

[59] Larsen M Rantala R Koudenburg OA Gulis G. Intersectoral action for health: The experience of a Danish municipality. Scand J Public Health. (2014) 42:649–57. 10.1177/1403494814544397 25074270

[60] Lillefjell M Magnus E Knudtsen MS Wist G Horghagen S Espnes GA. Governance for public health and health equity: the Tröndelag model for public health work. Scand J Public Health. (2018) 46:37–47. 10.1177/1403494818765704 29862906

[61] Bhatia R Corburn J. Lessons from San Francisco: health impact assessments have advanced political conditions for improving population health. Health Aff. (2011) 30:2410–8. 10.1377/hlthaff.2010.1303 22147870

[62] Freire MSM De Sá RMPF Gurgel IGD. Healthier sairé: a intersectorial policy as a turning point for local equity. Cien Saude Colet. (2017) 22:3893–902. 10.1590/1413-812320172212.25052017 29267707

[63] Hagen S Torp S Helgesen M Fosse E. Health in all policies: a cross-sectional study of the public health coordinators' role in Norwegian municipalities. Scand J Public Health. (2015) 43:597–605. 10.1177/1403494815585614 25975671

[64] Holt DH Rod MH Waldorff SB Tjørnhøj-Thomsen T. Elusive implementation: An ethnographic study of intersectoral policymaking for health. BMC Health Serv Res. (2018) 18:54. 10.1186/s12913-018-2864-9 29378655 PMC5789672

[65] Holt DH Waldorff SB Tjørnhøj-Thomsen T Rod MH. Ambiguous expectations for intersectoral action for health: a document analysis of the Danish case. Crit Public Health. (2018) 28:35–47. 10.1080/09581596.2017.1288286

[66] Mannheimer LN Gulis G Lehto J Östlin P. Introducing Health Impact Assessment: an analysis of political and administrative intersectoral working methods. Eur J Public Health. (2007) 17:526–31. 10.1093/eurpub/ckl267 17213235

[67] Pettman TL Armstrong R Pollard B Evans R Stirrat A Scott I. Using evidence in health promotion in local government: contextual realities and opportunities. Health Promot J Aust. (2013) 24:72–5. 10.1071/HE12902 23575594

[68] Storm I Harting J Stronks K Schuit AJ. Measuring stages of health in all policies on a local level: The applicability of a maturity model. Health Policy. (2014) 114:183–91. 10.1016/j.healthpol.2013.05.006 23764153

[69] Synnevåg ES Amdam R Fosse E. Legitimising inter-sectoral public health policies: a challenge for professional identities? Int J Integr Care. (2019) 19:4. 10.5334/ijic.4641

[70] Van Vliet J. How to apply the evidence-based recommendations for greater health equity into policymaking and action at the local level? Scand J Public Health. (2018) 46:28–36. 10.1177/1403494818765703 29862905

[71] Grimm MJ Helgesen MK Fosse E. Reducing social inequities in health in Norway: Concerted action at state and local levels? Health Policy. (2013) 113:228–35. 10.1016/j.healthpol.2013.09.019 24168889

[72] Marks L Hunter DJ Scalabrini S Gray J McCafferty S Payne N. The return of public health to local government in England: Changing the parameters of the public health prioritization debate? Public Health. (2015) 129:1194–203. 10.1016/j.puhe.2015.07.028 26298589

[73] Kneale D Rojas-García A Thomas J. Obstacles and opportunities to using research evidence in local public health decision-making in England. Health Res Policy Syst. (2019) 17. 10.1186/s12961-019-0446-x 31248422 PMC6598344

[74] McGill E Egan M Petticrew M Mountford L Milton S Whitehead M. Trading quality for relevance: non-health decision-makers' use of evidence on the social determinants of health. BMJ Open. (2015) 5:e007053. 10.1136/bmjopen-2014-007053 25838508 PMC4390684

[75] Bekken W Dahl E Van Der Wel K. Tackling health inequality at the local level: Some critical reflections on the future of Norwegian policies. Scand J Public Health. (2017) 45:56–61. 10.1177/1403494817701012 28850009

[76] Browne GR Davern M Giles-Corti B. What evidence is being used to inform municipal strategic planning for health and wellbeing? Victoria, Australia, a case study. Evid Policy. (2017) 13:401–16. 10.1332/174426416X1465565506200023599727

[77] Stoneham M Dodds J. An exploratory study identifying where local government public health decision makers source their evidence for policy. Health Promot J Aust. (2014) 25:139–42. 10.1071/HE14012 25200469

[78] Willmott M Womack J Hollingworth W Campbell R. Making the case for investment in public health: Experiences of Directors of Public Health in English local government. J Public Health. (2016) 38:237–42. 10.1093/pubmed/fdv035 25775932 PMC4894482

[79] South E Lorenc T. Use and value of systematic reviews in English local authority public health: a qualitative study. BMC Public Health. (2020) 20:1100. 10.1186/s12889-020-09223-1 32660533 PMC7359488

[80] de Goede J van Bon-Martens MJH Putters K van Oers HAM. Looking for interaction: quantitative measurement of research utilization by Dutch local health officials. Health Res Policy Syst. (2012) 10:9. 10.1186/1478-4505-10-9 22414224 PMC3341192

[81] Hagen S Øvergård KI Helgesen M Fosse E Torp S. Health promotion at local level in Norway: The use of public health coordinators and health overviews to promote fair distribution among social groups. Int J Health Policy Manag. (2018) 7:807–17. 10.15171/ijhpm.2018.22 30316229 PMC6186475

[82] Browne GR Davern M Billie GC. ‘Punching above their weight': a qualitative examination of local governments' organisational efficacy to improve the social determinants of health. Aust N Z J Public Health. (2019) 43:81–7. 10.1111/1753-6405.12847 30457190

[83] Collins PA. Do great local minds think alike? Comparing perceptions of the social determinants of health between non-profit and governmental actors in two Canadian cities. Health Educ Res. (2012) 27:371–84. 10.1093/her/cys009 22319077

[84] Synnevåg ES Amdam R Fosse E. Public health terminology: hindrance to a Health in All Policies approach? Scand J Public Health. (2018) 46:68–73. 10.1177/1403494817729921 28927351

[85] Didem EE Filiz E Orhan O Gulnur S Erdal B. Local decision makers awareness of the social determinants of health in Turkey: a cross-sectional study. BMC Public Health. (2012) 12:437. 10.1186/1471-2458-12-437 22703525 PMC3461478

[86] Guldbrandsson K Fossum B. An exploration of the theoretical concepts policy windows and policy entrepreneurs at the Swedish public health arena. Health Promot Int. (2009) 24:434–44. 10.1093/heapro/dap033 19819897

[87] Fisher M. Challenging Institutional Norms to Improve Local-Level Policy for Health and Health Equity: Comment on “Health Promotion at Local Level in Norway: The Use of Public Health Coordinators and Health Overviews to Promote Fair Distribution Among Social Groups”. Int J Health Policy Manag. (2018) 7:968–70. 10.15171/ijhpm.2018.67 30316252 PMC6186467

[88] Baldwin L Dallaston E Bennett B McDonald F Fleming ML. Health in all policies for rural and remote health: a role for Australian local governments? Aust J Public Adm. (2021) 80:374–381. 10.1111/1467-8500.12460

[89] De Blasio A Girán J Nagy Z. Potentials of health impact assessment as a local health policy supporting tool. Perspect Public Health. (2012) 132:216–20. 10.1177/1757913910391039 22991368

[90] Lowe M Whitzman C Badland H Davern M Aye L Hes D. Planning healthy, liveable and sustainable cities: How can indicators inform policy? Urban Policy Res. (2015) 33:131–44. 10.1080/08111146.2014.100260635561726

[91] Baker P Friel S Kay A Baum F Strazdins L Mackean T. What enables and constrains the inclusion of the social determinants of health inequities in government policy agendas? A narrative review. Int J Health Policy Manag. (2018) 7:101. 10.15171/ijhpm.2017.130 29524934 PMC5819370

[92] Baum F Graycar A Delany-Crowe T De Leeuw E Bacchi C Popay J. Understanding Australian policies on public health using social and political science theories: reflections from an Academy of the Social Sciences in Australia Workshop. Health Promot Int. (2019) 34:833–46. 10.1093/heapro/day014 29684128

[93] Battel-Kirk B Chiou ST Comeau L Dillon R Doherty K Jones-Roberts A. The IUHPE Health Promotion Accreditation System – developing and maintaining a competent health promotion workforce. Glob Health Promot. (2021) 28:46–50. 10.1177/17579759211029603 34308711

[94] Lucyk K McLaren L. Taking stock of the social determinants of health: a scoping review. PLoS ONE. (2017) 12:e0177306. 10.1371/journal.pone.0177306 28493934 PMC5426664

[95] Jones MD Peterson HL Pierce JJ Herweg N Bernal A Lamberta Raney H. A river runs through it: a multiple streams meta-review. Policy Stud. (2016) 44:13–36. 10.1111/psj.12115

[96] Pierce JJ Peterson HL Jones MD Garrard SP Vu T. There and back again: a tale of the advocacy coalition framework. Policy Stud. (2017) 45:S13–46. 10.1111/psj.12197

