# Supplementary material for: Factors of the policy process influencing Health in All Policies in local government: A scoping review
Source: Front Public Health. 2023 Feb 9;11:1010335. doi: 10.3389/fpubh.2023.1010335 (PMC9949293; doi:10.3389/fpubh.2023.1010335)
Supplement: Supplementary file 1 [file Data_Sheet_1.pdf]

Study citation	Year	Country	Level of Gov	Key concept of focus	Aim / objective of the research	Research methods	Research participants	Applied theory	Application of PST?	How applied PST?	Key findings - barriers and/or enablers identified in the policy process	Other key findings in the source	Comparison of different LG context/jurisdictions
Exworthy, M., Berney, L., & Powell, M. (2002). 'How great expectations in Westminster may be dashed locally': the local implementation of national policy on health inequalities. Policy & Politics, 30(1), 79-96.	2002	UK	Local	Health inequalities	Aim to describe how health inequalities gets onto the local and national policy agendas and how it is being actioned.	3 case studies (rural, urban and mixed suburbs): incl. interviews, documentation and meeting observations.	Majority local and some national policymakers.<br>Senior managers, Directors of Planning, Public Health doctors, managers in HA's (Health Authorities and networks, including local authorities, NHS, voluntary sector).<br>Method does not specify how many interviews were completed.	MSF	Yes	Interpret the findings in the discussion.	Enabler: Having national policy explicitly refer to health inequalities gave local legitimacy to address this issue.<br>Enabler: Supported policy by the 1997 Labour government<br>Barrier: 'Crowded' policy agenda, health inequalities not being prioritised amongst other local and national issues<br>Barrier: National expectations of local government were not met - largely reported as a lack of structures in place for 'Joined up government'. Policies were then not technically feasible.<br>Barrier: There were not the performance measures for health inequality, whereas local agencies reported to national government things like waiting lists and organisational finances. Any performance measures for health inequalities were 'soft' and vague.<br>Barrier: Action on health inequalities is reliant on policy entrepreneurs.	Application of MSF:<br>Problem, Policy and Politics streams had been partially coupled at national and local levels. The problem was on the agenda, some actions were taken and politics 'uncontroversial'. Howeveer there were issues. At a local level, health inequalities was not seen as an urgent problem, did not have performance monitoring targets attached to it and was less a priority than other issues such as waiting lists. Within the politics stream, the national policy helped shape local policy, but was largely left to policy entrepreneurs to manage. Policy stream still developing, with some action across agencies, but may be difficult to sustain over the long term, particularly if it continues to be seen as a 'health' problem.	Wasn't raised (even though case study sites chosen for this reason)
Bagley, P., Lin, V., Keating, T., Wise, M., & Sainsbury, P. (2007). In what ways does the mandatory nature of Victoria's municipal public health planning framework impact on the planning process and outcomes?. Australia and New Zealand Health Policy, 4(1).	2007	Australia	Local	Public health	Aim to identify the strengths and weaknesses of the implementation of mandatory municipal public health framework in Victoria.	Qualitative semi-structured interviews	10 management staff involved in public health planning in Victorian LG, plus 4 interviews with state-based public health staff.	None	No		Enabler: Majority agreed that legislation improved planning. Some reported that it increased the visibility of public health, although others saw it as another responsibility passed on by the State government.<br>Barrier: Significant variation exists: Different LG's approached the process in different ways e.g. in selecting priorities and engaging community. There does not seem to be a shared understanding of the nature of the MPHP.<br>Enablers: Factors such as management support, organisational culture, community expectations and the skills of staff were also relevant to how the process of MPHP was undertaken.<br>Barrier: Focus on process: Only half of the councils had an implementation plan for their MPHP. The process appears strategic, moreso than operational for some councils.<br>Barrier: Tension over priorities: Staff may identify local priorities, but be overridden with political decisions and demands, or community pressure. Where LG have specific legislation (e.g. food safety), these get higher priority than other public health activities and actions.	Respondents raised the degree of wariness regarding 'cost shifting' by State and Federal government, with responsibility for public health increasing though not with the appropriate level of funding required.<br>Regulation is not enough for effective public health planning in LG, although may act as an influencing factor.<br>Lessons learned: Legislative change needs to be consultive with LG, the scope should be clear, senior management support is critical, capacity of the workforce needs to be considered in planning, integration of LG and State planning is required, investment needs to be in implementation as well as development.	There were no clear differences in council size. Those that took a coordinated and social model approach to the plan were not always the wealthiest of councils and those that took a narrow approach were not always the less financially resourced.
Hoeijmakers, M., De Leeuw, E., Kenis, P., & De Vries, N. K. (2007). Local health policy development processes in the Netherlands: an expanded toolbox for health promotion. *Health Promotion International*, *22* (2), 112-121.	2007	Netherlands	Local	Determinants of health	Aim to learn why health policy often fails in LG by using stakeholder and network analysis.	Semi structured interviews (no number specified)<br>Observation<br>Document analysis	Based on 4 municipalities that were commencing local health policy development.<br><br>Included stakeholders in both professional and non-professional organisations.	MSF and Network analysis	Yes	The study utilises the network analysis very clearly. This was used to map stakeholders in the policy process and describe their characteristics. This formed the basis for the results of the information collected. Also formed the interpretation of the data in the discussion.<br>The MSF is not applied in full – only looking at the role of the policy entrepreneur.		Found that actors don't engage in strategic networking for health issues, but for own agendas.<br>Local health policy was not important to stakeholders.<br>Whilst the PHS (regional health service) were closely communicating and strategically engaging with municipalities in the networks, they were more removed in action.<br>Municipalities were 'central' to networks in all of the 3 networks mapped. However, this does not mean that they acted as an entrepreneur. Others expected them to act as the role of facilitator.<br>Municipalities seemed to be overwhelmed with the wide scope of health included in local health policy.<br>Community groups and organisations were not as closely networked.<br>There was no evidence of a policy entrepreneur.<br>Findings: There are no policy entrepreneurs acting at a local level, there is limited importance put on public health by stakeholders (and closely networked organisations tended to be healthcare oriented), and stakeholders engage for their own interests.	Nil

Mannheimer, L. N., Gulis, G., Lehto, J., & Östlin, P. (2007). Introducing Health Impact Assessment: an analysis of political and administrative intersectoral working methods. *European Journal of Public Health*, *17* (5), 526-531.	2007	Slovakia	Local	HiAP	Aims to identify the enablers and barriers to adopt a new way of working horizontally and intersectorally using HIA.	Qualitative Interviews.<br>Doesn't state how many interviews were conducted.	Civil servants and directors, politicians, researchers, representative of local public health institute - who were all involved in the pilot of a HIA approach.	MSF	Yes	MSF used to analyse and interpret the findings.	Enabler: Politicians showed commitment and political will for HIA.<br>Enabler: HIA was seen as a legitimate approach by the WHO and international organisations advocating the approach at the time.<br>Enabler: The local government and university had developed linked with the WHO e.g. membership to the WHO Healthy Cities programme. This also opened a window of opportunity to draw attention to HIA.<br>Barrier: Lack of formal cooperation between departments.<br>Barrier: HIA not institutionalised or a formalised process.<br>Barrier: Political commitment to HIA was not matched with sufficient resources to implement.<br>Barrier: Civil servants did not know if data was available or what to do with it.<br>Barrier: Civil servants found it difficult to start HIA processes in real context.	The main 'problem' that initiated action was the poor health status of the Slovakian population, combined with lack of intersectoral collaboration - which opened a window of opportunity.<br>Changes to the way Slovakia was being governed (in a major overhaul from authoritarian-egalitarian to liberal-democratic) changed the way health was defined and administrative functions.<br>There were few external stakeholders or public involved.	Nil
Guldbrandsson, K., & Fossum, B. (2009). An exploration of the theoretical concepts policy windows and policy entrepreneurs at the Swedish public health arena. Health promotion international, 24(4), 434-444.	2009	Sweden	Local	Child health promotion	Aim to identify if the concepts of 'policy window' and 'policy entrepreneur' could be empirically evidenced within existing policy cases.	Qualitative interviews and document analysis. Nine measures related to child and youth health across 3 municipalities.	50 interviews with municipality staff, managers and politicians; along with NGO representatives. Documents included action plans, politicial meeting minutes, evaluations, NGO documents.	MSF	Yes	Various political science theories and frameworks informed the interview guide. The MSF (specifically policy windows and policy entrepreneurs) formed the basis for data analysis, results and discussion.		In 8 of the 9 cases included, there was evidence of policy, problem and politics streams that opened a policy window.<br>There was evidence of different types of policy entrepreneurs in all cases, politicians that could speak for others and make a clam in a position of decision making power (7 of 9 cases); LG staff with political connections and negotiation skills (8 of 9 cases); 'sheer persistence' evidenced in 8 of 9 cases - including both politicians and staff.<br>Conclude that being able to better predict the opening of policy windows would make public health decision making more 'straightforward', along with 'storing' policy solutions ready for when the problem and politics streams allow the window to open.	Nil
Jansson, E. V., & Tillgren, P. E. (2010). Health promotion at local level: a case study of content, organization and development in four Swedish municipalities. *BMC Public Health*, *10* (1), 1-12.	2010	Sweden	Local	Health promotion	Aim to understand the processes in the initiation, development and organisation (including type and content) of health promotion in Swedish municipalities between the 1980's and 2006.	Case study:<br>Four municipalities were purposely chosen to be of similar geographical area, though different internal leadership and governance	Document content analysis and interviews (n=36)<br>Interviews included 30 officials (staff) and 6 politicians.	MSF and Social Change Theory	Yes	Introduced (briefly) social change theory and MSF in the introduction to the article. This was not revisited in the methodology, results or discussion. The discussion did explicitly identify that the study purposely took an inductive approach.	Enablers:<br>Internal initiators: local issues, champions (particularly those in power), local events, stable leadership.<br>External initiators: national recommendations, funding, statistics on locality, media reports and "to some extent" general societal health trends.<br>The external pressures of national health policy are most likely to create action where there is a local champion.<br>Internal factors were seen as more powerful initiators than external factors.<br><br>Barriers:<br>The implementation (action) needs to be supported by management and politicians.<br>The lack of explicit governance for health promotion in municipalities was seen as a challenge – everyone agrees it is their responsibility but where the accountability laid was unclear	Health promotion became more formalised for 2 case study sites where there were 'champions' with interest in health promotion.<br>Cross – department action was dependent on how clear the objectives and measures were for public health action in the operational planning.<br>Content/achievements – whilst initiating and developing health promotion were more conscious choices, outputs were not always intentional or "conscious" strategic choices.<br>Municipalities did not necessarily follow national strategic directions (that were more behaviour focussed).<br>Increasingly limited finances at the municipality level increased the reliance on working with other sectors to achieve action.<br>Whilst there were limited measurable goals, this did not inhibit action.<br>Some level of collaboration was developed with external sectors, across all four municipalities.<br>Over the years, there has been a shift in focus from a behavioural approach to a more holistic view, addressing structural determinants of health.	Nil
Schmidt, M., Joosen, I., Kunst, A. E., Klazinga, N. S., & Stronks, K. (2010). Generating political priority to tackle health disparities: a case study in the Dutch city of The Hague. *American Journal of Public Health*, *100* (S1), S210-S215.	2010	Netherlands	Local	Health inequities	Aim to determine facilitating factors to gaining political priority of health disparities within The Hague municipality.	Single case study design including semi-structured interviews, document analysis and observations. Prospective study, based on a 4 year research program.	14 people interviewed (the program leader interviewed 23 times). Other participants included councillors, managers and policymakers. Documents included program documentation, meeting minutes, political documents. Observed 17 project steering group meetings.	Shiffman and Smith	Yes	Framework by Shiffman and Smith used for data analysis (selective coding), which framed the subheadings for the results incl. actors, framing and political context.	Enabler: Committed councillors willing to tackle health disparities, based on their political ideology.<br>Barrier: Framing of health inequities as 'unfair' gained little political attention and support.<br>Enabler: Reframing the issue (e.g. agreeing with the debate that individuals were responsible for their own health, though highlighting how environmental structures made this difficult.), linking the issue to visions of the local municipality (e.g. all of us together).<br>Enabler: Presenting disaggregated data, gaining credibility to the issue.<br>Enabler: Framing solutions to existing policies that were already recieiving political attention.<br>Enabler: Involving academic researchers working on health equity research programs.<br>Enabler: Supporting the program goal of engaging and involving citizens in policymaking, which received attention by the municipality executive team.		Nil

Bhatia, R., & Corburn, J. (2011). Lessons from San Francisco: health impact assessments have advanced political conditions for improving population health. *Health Affairs* , *30* (12), 2410-2418.	2011	USA	Local	Determinants of health	Focus on how HIA affect broader political conditions for health.	Not stated: Based on experience of the authors.	"synthesis" of communication and dialogue with public and private organisational stakeholders and decision makers and public involved in HIA, along with government documents, media reports and personal observations.	None	No		Barrier: Planners were concerned that doing a health impact assessment (in addition to an Environmenttal Health Assessment) would increase the timeframes and budget.<br>Barrier: The validity of the HIA was often questioned, considered 'speculative'.<br>Barrier: HIA is not institutionalised into any systematic approach within LG<br>Enabler: Health staff limited involvement in EHA's and instead focussed on plans and actions that could influence health.<br>Enabler: HIA has likely increased awareness of the social determinants of health amongst stakeholders and the community, sustained for use across other projects.<br>Enabler: Health staff conducting HIA's were further trained and skilled in assessing possible health impacts (e.g. how to apply epi data and do risk assessments)<br>Enabler: The process of conducting a HIA provided opportunities for learning across multiple stakeholder groups and helped strengthen existing advocacy coalitions.<br>Enabler: Engaging with policy planners and stakeholders has helped health staff learn more about how to engage in the policy process and influence different policy sectors.<br>Enabler: Completing HIA built trust and working relationships between different stakeholders and the health department, allowing for more communication and information sharing.	HIA is technical as well as political. HIA may only be effective when it is aligned to the political environment.	Nil
Collins, P. A. (2012). Do great local minds think alike? Comparing perceptions of the social determinants of health between non-profit and governmental actors in two Canadian cities. Health Education Research, 27(3), 371-384.	2012	Canada	Local	SDoH	Aim to analyse the perceptions of SDOH amongst staff in community-based organisations and local municipalities	Mail administered survey.<br>The two surveys were conducted separately – CBO's in 2003 and GOV (municipalities) in 2008.<br>CBO survey in Hamilton, Ontario. GOV survey in Vancouver.	Responses include 241 CBO, and 345 GOV participants, response rates 55% and 54% respectively.<br>CBO participants mostly volunteers.<br>GOV participants mostly city staff.<br>Approx half of respondents in each sample held positions of power e.g manager.	None	No		Enabler: Both GOV and CBO participants identify that they have influence over living conditions of populations.<br>Barrier: Structural determinants of health were given the least priority by both CBO and GOV participants.<br>Barrier: Lack of access to resources to take action.<br>Barrier: Level of priority given to healthcare.	Both CBO and GOV participants rated 'healthy lifestyles' and 'clean air and water' as having a high level of influence over health. These determinants were also rated as the highest priority.<br>The lowest level of priority was given to 'income'.<br>Both CBO and GOV participants were similar for most of the determinants.<br>Perception of influence did not match level of priority. The determinants of 'income', 'social supports' and 'healthy lifestyles' were reported as having a higher level of influence over health than priority given. Contrary to this, 'healthcare' was assigned less influence compared to priority.<br>The high level of priority for determinants such as 'clean air and water' were given for different reasons – with CBO respondents from a heavily polluted region, and GOV respondents largely responsible for this in their role in local gov.<br>Conclude that shared understandings between CBO and GOV participants increases likelihood of collaborations, the priority would likely be given to healthy lifestyles that have minimal influence on health equity.	Nil
de Blasio, A., Girán, J., & Nagy, Z. (2012). Potentials of health impact assessment as a local health policy supporting tool. *Perspectives in public health* , *132* (5), 216-220.	2012	Hungary	Local	Health inequity	Aim to describe experience of using HIA as a 'health filter' in one city, a designated healthy city in Hungary (Pecs).	Case study (narrative): Pecs (a designated Healthy City)		None	No		Barrier: Given there was no legal obligation to do a HIA, there was limited motivation. Other barriers were short timeframes, a weak preparation phase (which led to officials not being confident to fulfill the role of HIA in decision making)and lack of support from political decision makers	Description of the attempts to systemise HIA as part of a plan to address determinants of health in a healthy city.<br>Specialist training was provided to 32 officials of different departments within the city administration. This was based on the already available human resources that were working within the policy decisions and had a good understanding of the operation of the council.<br>HIA is not only a technical component of decision making, though requires high level political support.	Nil
de Goede, J., van Bon-Martens, M. J., Mathijssen, J. J., Putters, K., & van Oers, H. A. (2012). Looking for interaction: quantitative measurement of research utilization by Dutch local health officials. Health Research Policy and Systems, 10(1), 1-12.	2012	Netherlands	Local	Public health	Aim to understand how local health officials use epidemiological research and identify the factors that influence this use.	Online questionnaire to possible 339 local health officials. Initial contact by phone for consent to participate.<br><br>Data analysed using linear regression model (Likert data treated as interval data)	155 local health officials reponsible for the development of local health policy. Representing 35% of municipalities.	Conceptual framework on research utilisation (authors developed), Use of research by Amara et al: Instrumental, Conceptual or Symbolic.	No		Enabler: Involvement of local officials in the research process was related to instrumental and symbolic use of research.<br>Unknown explanation: Unawareness of media publication about epidemiological data related to less instrumental use of research.<br>Enabler: Epidemiologist presenting the data via a presentation (rather than another staff member) related to conceptual use of research. Although this decreased as score for 'barriers to interaction' increased.	Research use:<br>Conceptual - better understanding and awareness<br>Instrumental - direct application of research into action<br>Symbolic - use to justify action or lack of action<br><br>Use of research was more commonly used for conceptual use, rather than instrumental or symbolic use.<br>This supports the complexity of the use of evidence-based research in policy, as local officials also deal with other actors, opinions and interests.	Nil

Didem, E. K. E., Filiz, E., Orhan, O., Gulnur, S., & Erdal, B. (2012). Local decision makers' awareness of the social determinants of health in Turkey: a cross-sectional study. *BMC Public Health*, *12* (1), 1-9.	2012	Turkey	Local	SDoH	Aim to look at awareness of social determinants of health amongst decision makers in the Aydin Province, Turkey.	Questionnaire (faxed to 53 Mayors and done as interviews face to face with 22 Headman)	50 Mayors, 22 Headmen	None	No			Mayors were aware of SDoH, though they were unsure how to action this in practice. Headmen were not aware of SDoH and equally unable to determine how to action this in practice.	Nil
Spiegel, J., Alegret, M., Clair, V., Pagliccia, N., Martinez, B., Bonet, M., & Yassi, A. (2012). Intersectoral action for health at a municipal level in Cuba. *International journal of public health*, *57* (1), 15-23.	2012	Cuba	Local	Determinants of health	Aims to determine how models of intersectoral action for health across municipal governments help explain health achievements in Cuba.	Mixed method design in two phases. Phase one: Questionnaires (pre-focus group) and focus groups in 2 municipalities (one city and one rural) Phase two: Indepth interviews with municipal staff on specific policy case studies (e.g. healthy children)	Municipal staff, healthcare sector, non-health sectors and community organisations.	None	No		Enabler: The local level primary health services (polyclinics) reinforces a 'place-based' approach to health. Enabler: The organisational structures generally were very formal and well established, including 'Health Councils' that engaged with non-health sectors at a local level. There are multiple intersectoral committees existing at the municipal level. Enabler: There is a high level of political support in Cuba for health-oriented outcomes.	Engaging with other sectors on a regular and consistent basis was a common theme in both municipalities studied. Intersectoral collaboration was strongest for programmes related to healthy lifestyles, when compared to healthcare or healthy child development programmes.	Intersectoral collaboration amongst all determinants was higher in the urban municipality than the rural municipality. Specifically for determinants such as gender and social supports.
Steenbakkers, M., Jansen, M., Maarse, H., & de Vries, N. (2012). Challenging Health in All Policies, an action research study in Dutch municipalities. *Health policy*, *105* (2-3), 288-295.	2012	Netherlands	Local	HiAP	Aim to determine the effectiveness of a coaching program in HiAP with municipalities in the Dutch region.	Participatory action research over 30 months. Internet questionnaire pre and post + interviews with stakeholders. Log book of all activities.	32 municipalities were involved in the research. 9 volunteered to be in the coaching program. 13 indepth interviews with Public Health managers in municipalities (8 within the coached sites)	None	No			Coached municipalities showed greater outcomes in HiAP than non-coached. Interventions related to obesity prevention increased in the coached municipalities – but their intentions to continue this in the future decreased. At a strategic level, political priority for HiAP decreased in the coached municipalities. At a tactical level, manager support for HiAP decreased in the coached municipalities. At an operational level, no difference in knowledge, attitudes, self efficiacy of perceived expectations towards HiAP between coached and non-coached municipalities. Findings: Intersectoral action requires management support, health might not be the primary goal but community-oriented such as social cohesion.	Nil
Collins, P. A., & Hayes, M. V. (2013). Examining the capacities of municipal governments to reduce health inequities: a survey of municipal actors' perceptions in Metro Vancouver. *Canadian Journal of Public Health*, *104* (4), e304-e310.	2013	Canada	Local	Health inequities	Aim of the study to measure understanding of SDoH, perceived role of LG, policies that could impact on HI, and barriers to addressing HI.	Mail survey to 637 politicians and staff in 17 Municipal governments in Metro Vancouver.	345 participants, 56% non-elected officials and 54% elected officials.	None	No		Barriers:little empowerment to reduce HI with little autonomy, insufficient funding from senior governments, insufficient collaboration with higher tiers of government and reluctance to increasing property taxes to increase funding.<br><br>Enabler: Study demonstrated respondents were willing to discuss HI, even if limited perceived power to influence HI.	There were no consistent differences in responses based on role or sector within a municipality. Understanding of SDoH – wasn't answered directly. Respondents saw that provincial and federal governments had the most responsibility for HI. Individuals were 4th – suggesting that there still exists a sense of responsibility by individuals to act (more than municipal government responsibility). Of all respondents, 44% reported a high priority to addressing HI. The highest priority areas were given to parks and recreation facilities, community centres and citizen engagement – as ways for municipalities to address HI. Property taxes were given the least priority. Respondents identified a broad range of policies, plans and programs that address HI in their jurisdictions. Homelessness and affordable housing were most commonly identified, following by fitness, parks and recreation programs.	Non-metro council respondents were less familiar with the broad determinants of health. Results indicated the respondents viewed the sectors responsible for addressing HI in the following order (highest to lowest): provincial government, federal government, regional health authorities, individuals, regional governments, municipal governments, non-profit sector, market. Authors suggested this ranking may reflect participant concerns about downloading of responsibilities from higher to lower levels of government.

née Grimm, M. J. T., Helgesen, M. K., & Fosse, E. (2013). Reducing social inequities in health in Norway: Concerted action at state and local levels?. Health policy, 113(3), 228-235.	2013	Norway	Local & National	Social inequities	Aim to see if local municipalities action the public health policies intended by the national public health act, explore the understanding of health among different policy sectors, and to explore the role of public health coordinators and resources in HiAP.	Includes 2 datasets:<br>Qual: content analysis of national level documents and interviews<br>Quant research: electronic questionnaires to a possible 430 local municipalities	Interviews with staff in Directorate of Health<br>Online survey with CEO of local municipalities (n=374)	None	No			Local municipalities – 6% explicitly consider underlying determinants of health<br>Most define challenges as drug abuse, mental health, nutrition and physical activity. Data suggests that municipalities prioritise behaviour and lifestyle related health issues.<br>Whilst required to do HIA's, 67% have not applied HIA's, 71% report a lack of health overviews.<br>Funding of municipalities supports the voluntary recruitment of public health coordinators. The intention of these coordinators is to establish partnerships to address health. 74% of municipalities have established positions. 46% of these are under the direction of a medical officer.<br>Most funding for health comes from within Local Municipalities. Some funding from national for 'earmarked' grants. National funding was not seen as a critical source by most municipalities.<br>Overall findings suggest that local municipalities have not adopted the intended public health act obligations set by the national government.	Nil
Pettman, T. L., Armstrong, R., Pollard, B., Evans, R., Stirrat, A., Scott, I., ... & Waters, E. (2013). Using evidence in health promotion in local government: contextual realities and opportunities. Health Promotion Journal of Australia, 24(1), 72-75.	2013	Australia	Local	Health promotion	Aim to describe and advocate for what is needed to plan and implement evidence-based health promotion in LG.	Facilitated discussions during group training sessions.	Academics and LG practitioners/staff in Victoria, Australia.	None	No		Enabler: There are LG staff capable and interested in evidence-informed health promotion practice.<br>Barrier: Horizontal cooperation is lacking across LG departments, particularly in larger councils.	What is needed:<br>Senior decision makers need to be supportive.<br>LG need internal champions for evidence-informed decision making.<br>LG need access to academic journal databases.<br>Suggestions for institutionalizing evidence-based health promotion is for 'prompts' in meeting agendas or 'toolkits' available for guiding use of evidence.	Horizontal cooperation is lacking across LG departments, particularly in larger councils.
Corburn, J., Curl, S., Arredondo, G., & Malagon, J. (2014). Health in all urban policy: city services through the prism of health. Journal of urban health, 91(4), 623-636.	2014	USA	Local	HiAP	Aim to describe the processes in the development of HiAP in Richmond, California.	25 interviews – leaders of LG and community-based organisations.<br>Document analysis of minutes, public meetings, emails, reports and other publicly available documents.<br>Used community surveys from 4 time points in 2007, 2009, 2011 and 2013 (resident perceptions of community wellbeing) (randomly administered to 3000 households).	Respondents were those in high level roles – some voluntary/paid.	None	No		Enabler: Initiation of a 30 year General Plan in LG - required by law with a section on 'community health and wellness element'<br>Enabler: "eventually" goals linked to HiAP strategy were costed in the LG budget.<br>Enabler: A HiAP leadership group was formed.<br>Enabler: Locally acquired data was used from community surveys.<br>Enabler: Ongoing community development work has increased the priority of health equity and created a 'value shift'.<br>Enabler: A community and government collaborative group is one of the positive outcomes of the process.<br>Enabler: Use of a 'cumulative toxic stress' model to identify the community health needs during community consultations - which demonstrated the multiple stress points that needed to be addressed (e.g. racism, violence, pollution) using an integrated approach - hence HiAP.<br>Enabler: Indicators were developed for each intervention area that could be used to monitor progress over time. Data had to be existing and publicly available.<br>Barrier: Reluctance of LG departments to be involved as they perceived it to add to their work.<br>Barrier: There was resistance from those in and outside of LG that did not share the values of environmental and social justice.<br>Barrier: Difficulty in getting local level health data (which made indicators difficult to measure).<br>The three key factors to healthy community development: Community involvement from the bottom up, coupled with LG support and willingness to engage; Pilot place-based actions – to engage community and see tangible changes; Learning by doing – building of partnerships that could apply for grants to continue the work.	Richmond has had persistent health inequities, with active citizens that have advocated for environmental and social justice for a long time.<br>"activists groups" advocated for environmental and social justice to be included in the General Plan.<br>The General Plan was the initiator of the process - a legally requried plan.<br>An early strategy was the formation of a collaborative partnership group called the Richmond Health Equity Partnership.<br>The LG applied and received a grant to incorporate a 'community health and wellness' component within the General Plan.<br>Community organisations and LG staff workshoped to narrow evidence of health equity research into six intervention areas that also aligned to LG General Plan and budget.<br>Community consultation was undertaken once the draft chapter of the plan was completed.<br>Evidence linked actions to health outcomes – included areas such as improved open space, access to healthy food, access to healthcare services, safe and active transport, affordable housing, economic opportunities, safety, health promotion services, sustainable development, improved environmental quality and government leadership.<br>Two short term, 'place-based' pilot projects developed from the draft plan. This pilot recognised gaps in the inclusion of schools, as well as integration of health into all decision making aspects of LG.	Nil
Dhesi, S. K. (2014). Exploring how Health and Wellbeing Boards are tackling health inequalities with particular reference to the role of environmental health. The University of Manchester (United Kingdom).	2014	England	Local	SDoH, focus on Environment al Health	Research Q#2: Do (and how) Health and Wellbeing Boards tackle health inequalities and does this vary between areas?	Qualitative case studies (n=4), longitudional approach using observation, interviews and document analysis.	31 case study site interviews with members of HWB boards.<br>19 interviews with EH managers and staff across different contextual sites (outside case study sites).<br>Observation of 23 HWB meetings.<br>Documents of meeting minutes, reference lists and HWB strategies.	Harrison's Design to Doodle conceptu al framewor k.	No		Enabler: Marmot report and objectives for addressing health inequities. Although the main priorities were issue-focussed, which gained the most consensus. Children and young people were also a priority.<br>Enabler: Shared commitment to addressing health inequities amongst HWB members.<br>Barrier: some sites saw health inequalities as a low priority, or outside of their control at a local level.<br>Barrier: Tools to address health inequities at a local level were seen to be lacking.<br>Barrier: The understanding of health inequities was not shared bween HWB members.<br>Barrier: The difficulty of measuring success of actions on health inequities.	Tensions were reported to exist between different policy actors e.g. officers, elected officials, GP's and different tiers of local authorities. Some debate was classified as healthy, where there were relationships built on trust. Some reported that these constructive debates were absent and created tensions within meetings (although this was not observed).	Nil

Larsen, M., Rantala, R., Koudenburg, O. A., & Gulis, G. (2014). Intersectoral action for health: the experience of a Danish municipality. *Scandinavian journal of public health* , *42* (7), 649-657.	2014	Denmark	Local	HiAP	Aim to determine the challenges and enablers to development and implementation of an intersectoral health policy in Varde, Denmark.	Single case study - used documents and semi-structured interviews (n=9).	Stakeholders involved in the development and implementation of the health policy across different municipal sectors.<br>Documents - Meeting minutes and working papers.	None	No		Barriers: silo departments make cross-sector policy difficult, seen as an extra task for staff, no funding (until a Fund for Health was established), lack of ownership, lacked clear objectives and performance indicators, unable to maintain political and public attention.<br>Enablers: local political support when developing policy (which decreased during implementation suggesting it is difficult to maintain the commitment), community participation and involvement, use of local media, information sharing through newly established Health Networks, set up of Fund for Health, having researchers involved – which interviewees perceived gave the policy higher priority, bringing people together in dialogue.	The benefits of intersectoral policy action can be difficult to measure and gain an understanding of the cost-benefit.	Nil
Morrison, J., Pons-Vigués, M., Bécares, L., Burström, B., Gandarillas, A., Domínguez-Berjón, F., ... & Borrell, C. (2014). Health inequalities in European cities: perceptions and beliefs among local policymakers. BMJ open, 4(5).	2014	Europe	Local	Health inequalities	Aim to determine public policymakers beliefs and perceptions of health inequalities in reference to policymaking.	Semi-structured interviews	19 public policy/decision makers across 13 cities, either elected councillors or senior non-elected officials. (Sample involved in the INEQ-Cities project). Health and non-health sectors.	None	No		Barriers: Organisational constraints/resistance from other levels of administration<br>Barrer: budget restrictions<br>Barrier: miscommunication with private sectors<br>Barrier: lack of awareness by individuals in society about healthy lifestyles was a barrier to addressing health inequalities.<br>Enabler: Access to data that highlighted health inequalities is more likely to see action to address them.<br>Enabler: Some respondents highlighted the opportunity of working with community groups to get access to 'hard to reach' groups.	There were mixed understandings of health inequalities. Most were aware of the concept and related this to differences in health as a result of various social determinants of health e.g. income, neighbourhood where they lived. A few did not understand health inequalities, believing that health outcomes are impacted by individual responsibilities.<br>Most reported that health inequalities was a priority - responded that addressing health inequalities was an objective of the city government. Two did not consider it to be a priority, one citing that it was not in their responsibility at a local level.<br>Most conduct or rely on regular surveys to measure health outcomes.<br>When asked about what policy actions are taken, most responded with individual level behavioural actions (e.g. smoking, poor diets).<br>Some had partnerships established for intersectoral collaboration, while others saw this as difficult to get things done.	Nil
Stoneham, M., & Dodds, J. (2014). An exploratory study identifying where local government public health decision makers source their evidence for policy. Health Promotion Journal of Australia, 25(2), 139-142.	2014	Australia	Local	Public health	The aim was to identify what categories/types of evidence were being used by local government staff to inform public health plans.	Online survey to 5 of 140 local governments in Western Australia. Chosen as they had commenced new public health plan (included 3 metro and 2 regional LG's).	49% RR (n=533).<br>Staff included were in professional roles eg. corporate services, community development, planning departments.	None	No			Respondents indicated that they used 1) observational data of risks they observed in the community (24.5%), 2) information in statewide plans (eg. statewide government plans (17.7%), 3) locally derived evidence from stakeholders and key community groups (17.6%) and 4) media sources (16.2%).<br>Others included organisational priority (6%), directorate priority (6%), hunch (5.2%), complaints and enquiries received (5.1%) and 'other' (not specified in article) (1.7%).<br>Authors recognise the risk of using media as a valid and reliable source of evidence.<br>Acknowledgement given for the limitations of time and availability of evidence that local government staff have readily accessible and that is locally applicable.	Nil
Storm, I., Harting, J., Stronks, K., & Schuit, A. J. (2014). Measuring stages of health in all policies on a local level: the applicability of a maturity model. *Health Policy* , *114* (2-3), 183-191.	2014	Netherlands	Local	HiAP	To determine if a maturity model can measure stages of HiAP growth.	Mixed method. Document analysis (health policy), Digital questionnaires (n=123, RR 79%) and interviews with policy officers in health, education, social affairs, planning and housing (n=32).<br>Of a possible 50 eligible municipalities (with activity in health inequities or HiAP).	Policy officers in health, education, social affairs, planning and housing (n=32). Across 16 different sized municipalities involved, with varying populations of advantage/disadvantage.	Various maturity models (management)	No		Enablers in early stages: Good relationships, positive experiences with intersectoral collaboration and shared interests.<br>Enablers in implementation/as progress: sufficient resources, sense of urgency to address health inequities, support by council and municipal councillors.		Nil

Fosse, E., & Helgesen, M. K. (2015). How can local governments level the social gradient in health among families with children? The case of Norway. International Journal of Child, Youth and Family Studies, 6(2), 328-346.	2015	Norway	Local	HiAP (focus on family and child health)	To explore if the national policy influences local level policy and how LG address social inequalities that influence services for family and children.	Two surveys (2011 and 2014) and focus groups in 6 municipalities.	CEO's and other relevant staff in the municipality.<br>2011 survey (n=361)<br>2014 survey (n=303)<br>Focus groups (n=	None	No		Barrier: National funding did not always match local priorities.	Development of a health overview increased from 13% to 24.5% between 2011-2014.<br>Many public health themes were raised in municipality plans (e.g. Long Term Master Plans) despite not having a health overview.<br>Established intersectoral working groups decreased from 95% in 2011 to 62% in 2014. Except for in areas of schools, CEO office and planning department, where there was an increase.<br>The employment of a public health coordinator increased from 74% (of 347 municipalities) to 85% (of 307) between 2011 and 2014.<br>In 2011, 43% of municipalities reported that health inequalities was a goal.<br>In 2014, 51% of municipalities reported they are capable of reducing health inequalities, identified as through strengthening welfare services and targeted measures for vulnerable groups.<br>Interviews support the approach to universal, as well as targeted measures to reduce social inequalities in economic plans.<br>Conclude that each municipality addresses social inequalities differently, based on how the problem is defined.<br>Conclude there needs to be explicit support from the National policy, including funding.	Nil
Hagen, S., Helgesen, M., Torp, S., & Fosse, E. (2015). Health in All Policies: A cross-sectional study of the public health coordinators' role in Norwegian municipalities. *Scandinavian journal of public health* , *43* (6), 597-605.	2015	Norway	Local	HiAP	Aim to describe baseline data of the use of public health coordinators in Norwegian municipalities prior to a new national, mandated public health act.	Used statistical data on municipalities from database sources, along with 2 online questionnaires distributed to all 428 Norwegian municipalities.	Surveys sent to CEO's and Admin Managers Good response rate from questionnaires 79% and 58%.	None	No			Municipalities with the following characteristics were more likely to employ and use a PHC:<br>• Collaborated with the county council<br>• Collaborated with private and voluntary sectors<br>• Developed a health overview<br>• Were of low median income<br>Factors not associated with employment of a PHC included:<br>• Size of municipality<br>• Political profile<br>• Revenues	Nil
Hendriks, A. M., Jansen, M. W., Gubbels, J. S., De Vries, N. K., Molleman, G., & Kremers, S. P. (2015). Local government officials' views on intersectoral collaboration within their organization–A qualitative exploration. *Health Policy and Technology*, *4*(1), 47-57.	2015	Netherlands	Local	Determinants of health	Aim to explore local government official's views on intersectoral collaboration – related to public health outcomes.	Method: 2 municipalities (small sized), 19 interviews with local officials across 10 different policy areas; observed meetings.	Local officials across policy areas	COM-B	No		Enabler: Understanding that most policy issues had multiple impacts, including health;<br>Enabler: Communication is an important skill.<br>Enabler: Motivations and emphasis on a good outcome for citizens<br>Barrier: Hierarchal structures create barriers;<br>Barrier: Different disciplines, described as 'soft' (more social services) and 'hard'(technical/tough legislative environment) had different views on how easy it would be to collaborate. Hard disciplines were more likely to resist change.	Public health officials need to directly approach other disciplines with ways their discipline area can support health outcomes.<br>Need to reframe the health problem to these disciplines.	Small municipalities were both positive and negative for intersectoral collaboration – positive as they tended to know one another, negative as resources and capacity can be tight in small municipalities.
Lowe, M., Whitzman, C., Badland, H., Davern, M., Aye, L., Hes, D., ... & Giles-Corti, B. (2015). Planning healthy, liveable and sustainable cities: How can indicators inform policy?. Urban policy and research, 33(2), 131-144.	2015	Australia	Local	SDoH	Aim to research current liveability indicators, and determine how these are used by decision makers to inform local health, liveability and sustainability policy.	Literature review of liveability indicators and consultation workshops.	Urban policy-makers, researchers, private and community-sector decision makers.<br>Workshop 1: 80 state and local government policy actors and planners<br>Workshop 2: 50 planners (mostly LG)<br>Workshop 3: academics and policy actors from all tiers of government.	None	No			Indicators were used to determine what the 'problem' was and why it is an issue; and also for monitoring trends over time.<br>Indicators were reported to be used for developing shared objectives across different departments.<br>It was felt that more 'neighbourhood-level' measures were needed to better inform policy, as well as to compare sub-population groups.<br>Measures of economic impact of policy decisions was also a felt need.<br>Indicators needed to be credible, easy to communicate and integrated into relevant policies and plans.	Nil

Marks, L., Hunter, D. J., Scalabrini, S., Gray, J., McCafferty, S., Payne, N., ... & Thokala, P. (2015). The return of public health to local government in England: changing the parameters of the public health prioritization debate?. Public health, 129(9), 1194-1203.	2015	England	Local	Public health	To identify the influence of values and contexts in priority setting for public health in local government, including the prioritisation of ring-fenced budgets.	Qualitative interviews (n=29)	22 Health and Wellbeing Board members, 4 public health staff, 1 elected member and 1 representative of the voluntary sector.	None	No		Influencing factors for prioritisation of public health investments:<br>Enabler: Organisational context - LG are accountable to local communities. Local autonomy given to local government allows them to set their own priorities.<br>Barrier: Priority setting models - difficulty in always agreeing on priority criteria e.g. short term wins for few vs long term gains for many. Much of the ring-fenced budget reflected healthcare service.<br>Barrier: Views of evidence – there is a tension and a balance needed between scientific evidence and local knowledge/political processes. Previously at a national level, traditional evidence-base was used, whereas local government value local knowledge.<br>Barrier: understanding of public health - issues raised where public health funding was small and meant different things to different local governments. At a national level of government, conflicts with healthcare budgets were the main concern. In local government, the concern was that budgets could be absorbed into other areas.<br>Directors of Public Health felt that more could be done to change organisational values around integrating health into decision making across the local authority.	A shift of public health responsibilities from national to local level government demonstrates the value of context in what influences priorities.	Nil
McGill, E., Egan, M., Petticrew, M., Mountford, L., Milton, S., Whitehead, M., & Lock, K. (2015). Trading quality for relevance: non-health decision-makers' use of evidence on the social determinants of health. *BMJ open* , *5* (4), e007053.	2015	UK, Brazil, USA, Canada	Local	SDoH	Aim to identify how information and evidence are defined and utilised by local decision-makers working in the built environment space.	Focus groups<br>2 in the UK<br>1 international (Brazil, USA, Canada)	15 senior local government decision makers, purposely selected in built environment roles.	None	No		Enabler: Access to local level data to compare with other local authorities or national indicators.	Health 'research' was seen as a 'pure science' in a biomedical model.<br>'Evidence' included a wide range of sources from routine data, GIS, anecdotes, case studies and academic research.<br>Being able to demonstrate 'viability' was very important. Interventions needed to align to national strategic objectives, politicians and the public.<br>Need to demonstrate viability through cost-effectiveness / value for money.<br>Decision makers also rely on personal local knowledge.<br>Local evidence that fits the local context was seen as far more important than rigorous academic research.	Nil
Morrison, J., Pons-Vigués, M., Díez, E., Pasarin, M. I., Salas-Nicás, S., & Borrell, C. (2015). Perceptions and beliefs of public policymakers in a Southern European city. *International journal for equity in health* , *14* (1), 1-10.	2015	Spain	Local	Health inequalities	To describe the beliefs and perceptions of public policymakers on healthy inequalities and the policies to reduce them.	Qualitative study: semi-structured interviews (n=12)	Local government decision makers (both elected and non-elected)	None	No		Barriers to policy implementation: Insufficient funding, opposition by the public, private society on some services or strategies.<br>Enabler to policy implementation: Bringing people together to collaborate.	All informants agreed that Barcelona experienced health inequalities.<br>Politicians tended to report health outcome differences (e.g. life expectancy). Others reported different aspects of health inequalities, social exclusion, effect of neighbourhoods etc. Non-health informants were aware of policies that indirectly impacted on health.<br>Politicians and officers went beyond a biomedical focus, with politicians more likely to report structural determinants as the cuase, whereas officers focussed on health behaviours and healthcare.<br>All politicians agreed health inequalities was a priority. It was felt that whilst not clearly defined, health inequalities is considered. The health officer and opposition party disagreed, reporting health inequalities was not a priority of the city council.<br>Most felt that health inequalities was part of the city council role, however informants from public health, welfare and education thought it was beyond the authority of local councils.<br>Politicians referred to an Annual Health Report, provided by the Barcelona Public Health Agency to gain information on health inequalities. Non-health officers considered that there is a lack of information.<br>All reported little intersectoral collaboration and coordination. Officers identified that they work with other sectors, usually around specific issues.	Nil

Phillips, G., & Green, J. (2015). Working for the public health: politics, localism and epistemologies of practice. *Sociology of health & illness*, *37* (4), 491-505.	2015	England	Local	Determinants of health	Aim to examine how LG officers make decisions that impact on determinants of health.	Organisational ethnography approach. 8 weeks of participant and non-participant observation in 6 local authority offices, including attending meetings, informal and formal conversations; using alochol policy as a case study.<br>Included range of city and county authorities.	LG officers	None	No		Enabler: Officers with built interpersonal relationships to mediate conflicting views and being able to manage multiple stakeholders.<br>Enabler: Officers who are able to balance the use of 'hard' data, with local knowledge, experience and intuition.	Health outcomes were not the primary goal, but could be an added benefit strategically to gain external funding or support other goals.<br>Emphasis in decision-making was given to the uniqueness of the local authority region (rather than just adopting best practice from elsewhere).<br>Multiple forms of evaluation are required, to balance community perceptions, accountability and organisational credibility. All actions had to be able to be defended in some way, so nothing could really 'not work'.<br>Concludes that decision making on actions in LG that impact on determinants of health requires a balance of local and national politics, public opinion and funding considerations.<br>Evidence-based paradigms that dominate the healthcare service were largely absent regarding LG addressing determinants of health.<br>Suggests that LG has always been managing population health and wellbeing, in the absence of medical public health specialists, which is a strength that could be focussed on further.	Nil
Langeveld, K., Stronks, K., & Harting, J. (2016). Use of a knowledge broker to establish healthy public policies in a city district: a developmental evaluation. *BMC Public Health*, *16* (1), 1-13.	2016	Netherlands	Local	Determinants of health	Aim to describe how the role of a knowledge broker worked at a local level.	Action research methodology, single case study site – city district within Amsterdam municipality (not quite the same as a constituted municipality structure).	Employees of the city district and the municipal public health service.<br>Researcher acted as the knowledge broker.	None	No		Enablers to integrating health into specific policy areas:<br>-Health already on the agenda of the policymaker.<br>-Creation of trust, ensuring conversations were confidential.<br>-Policy makers having a scientific or research background.<br>-Policies being broad-brushed.<br>-Suggestions that matched the level of responsibility of the district<br>-Knowledge broker had a strong background in policy agenda setting<br>-Presence of a knowledge broker increased awareness of health with other policy sectors,<br>-Support from middle-senior management and elected officials was built over time<br>Barriers to integrating health into specific policy areas:<br>-Knowledge broker not seen as a reliable source of information to make policy suggestions.<br>-Not building management support first.<br>-Each sector having their own specific responsibilities.<br>-Time constraints.<br>-Suggestions didn't match existing practice.<br>-Suggestions not matched with current goals.<br>-Suggestions considered too scientific or abstract.	Conclusion: Role of a knowledge broker was successful to integrate health into some policies, had an impact on organisational change to consider health more broadly across policies.	Nil
Storm, I., den Hertog, F., Van Oers, H., & Schuit, A. J. (2016). How to improve collaboration between the public health sector and other policy sectors to reduce health inequalities?–A study in sixteen municipalities in the Netherlands. International journal for equity in health, 15(1), 1-14.	2016	Netherlands	Local	Health inequalities	Aim to understand the level of collaboration between public health and other social and physical policy sectors.	Qualitative descriptive analysis, incl. document analysis, questionnaires and interviews.<br><br>16 of a possible 50 municipalities were included, all active in the field of HiAP.	Health policy documents at each municipality site.<br>Online questionnaires (n=98) with mostly policy workers in public health, social and physical policy sectors. Some program managers, policy developers, projct leaders or heads of department also responded.<br>Interviews (n=32) with policy workers in public health, education/youth affairs, sport, social affairs, housing/spatial planning (operational	None	No		Barrier: Addressing health equities was a low priority for staff in the physical policy sector, with lack of awareness of how their work was related and lack of clear objectives.<br>Enabler: Workers were more likely to consult across policy sectors to achieve a program outcome, or a shared area that they overlap, rather than work collaboratively to address an issue.<br>Enabler: Having a coordinator to support collaboration was reported important, reported by the public health sector as a role they played.<br>Enabler: Support from politicians and management was also important for collaboration (e.g. departmental managers and elected members) or through the encouragement of the municipality. Interviewees reported that the current support was more apparent in the social policy sector.	Social sectors include youth affairs, education and sport. Physical policy sectors include housing, spatial planning and environment.<br>Some municipality health policy documents addressed multiple determinants of health e.g. Addressing lifestyle, along with the social and physical environment. Interviewees report that this is not always with the intent to address health inequities.<br>Municipalities policy documents give less attention to social determinants of health e.g. unemployment.<br>Public health and the social policy sector worked together to explicitly address health inequities, in particular a large proportion of public health policy workers reported that they collaborate with sport policy staff (86%).<br>Public health were least likely to collaborate with physical policy sector.<br>Authors suggest the use of HiAP tools that are available to support health integration into social and physical policy sectors.<br>Recommendations also include more formal collaboration strategies, an explicit focus on health determinants early in collaborative discussions and gaining strategic, as well as operational support for collaborative action.	Nil

Willmott, M., Womack, J., Hollingworth, W., & Campbell, R. (2016). Making the case for investment in public health: experiences of Directors of Public Health in English local government. Journal of Public Health, 38(2), 237-242.	2016	England	Local	Determinants of health	Aim to identify what Directors of Public Health advocate for public health in local government, the components of their cases and what evidence they use for this.	Semi-structured telephone interviews for all 16 Directors of Public Health.	13 Directors of Public Health in LG	None	No		Enabler: Evidence was useful, synthesised evidence is useful, having a cost-effective argument was seen as important.<br>Barrier: having time to "sift" through the literature for good practice evidence.	The DPH identified 2 main issues that they need to advocate for, the control of the public health grant budget and to address broader determinants of health. The types of evidence used included peer review literature, examples of what other councils had done.<br>"evidence" in LG was seen as different than in public health generally – with the focus on making sure the case was a normative argument – in line with current priorities, doing the 'right thing' as well as being 'politically acceptable'. If this was the case, then other evidence was not required.<br>Any evidence needed to be made locally relevant by the DPHs. Telling local stories was also effective was also persuasive evidence.<br>The authors raise concern over the lack of evidence that the determinants of health has in terms of economic impact, and role of LG.<br>Authors confirm that evidence is only one part of the determinant of public policy.<br>More evidence on cost-effectiveness of action is required.	Nil
Bekken, W., Dahl, E., & Van Der Wel, K. (2017). Tackling health inequality at the local level: Some critical reflections on the future of Norwegian policies. Scandinavian Journal of Public Health, 45(18_suppl), 56-61.	2017	Norway	Local	HiAP	Aim to identify the 'opportunities and obstacles' for municipalities to take action on health inequalities.	Discussion: Government documents and research.	None	None	No		Barrier: National level policy support for addressing health inequities seems to be reducing over time. Authors argue that if national support for addressing health inequality does not continue, it makes the task of local level policy much more difficult to achieve outcomes.<br>Barrier: Many municipal level initiatives are not evaluated, or where they are they are not scientifically rigorous enough and may lack scientific and political influence/value.<br>Barrier: Poor access to local level data. It is the national government responsibility to provide this data to the local level, though if this is not fulfilled, it makes the task of local government knowing where to invest much more difficult (ie. No data, no problem…no problem, no action).	Translation of national policy to local policy requires a rework of the problem and solutions as seen from the perspective of local policy actors. Each municipality will respond differently to addressing health inequities, based on local adaptations.	Larger municipalities are more likely to have the resources, competence and organisational capacity to address health inequities.
Browne, G. R., Davern, M., & Giles-Corti, B. (2017). What evidence is being used to inform municipal strategic planning for health and wellbeing? Victoria, Australia, a case study. Evidence & Policy: A Journal of Research, Debate and Practice, 13(3), 401-416.	2017	Australia	Local	SDoH	Aim to describe the types of evidence that Victorian LG utilise to inform municipal health plans.<br>To explore sources of evidence, what evidence describes and how specific evidence is within documents.	Content analysis. Included: MPHP's or Community Plans of Victorian LGs – obtained from websites or council contacts.	LG documents. Included 116 documents across 79 LGs.	None	No		Barriers: Evidence as a challenge for LG, in Australian context, LG does not have the capacity to undertake reviews of evidence. Local evidence-informed intervention research is hypothesised as possibly non-existent, difficult to obtain, and where it is available and used it may not be cited.Where evidence does exist, it might not be appropriate for a local level.	Type of evidence categories: not specific, demographics, epidemiology, health behaviours, domains of public policy.<br>In 26% of occurrences, the source of evidence was unable to be determined.<br>ABS was the most highly cited source, followed by local council (eg. Community consultations) and State Government documents.<br>46% of evidence referred to 'domains of public policy' e.g. 17% referred to "social connectivity, cohesion and democracy".<br>Epi and behavioural health categories had highest citings related to mental health (18%) and nutrition (21%).<br>Majority of the evidence referred to the situation, rather than on evidence for action (4% of all occurrences).<br>Evidence for action was mostly 'council-generated', including via community consultation.<br>Academic sources were some of the least cited sources of evidence for evidence-based action.<br>It is possible that information is sought more informally through professional networks and colleagues.<br>A combination of peer-to-peer communication, informal networking and advice from population health experts is playing a role in MPHWPs.	Nil
Freire, M. D. S. M., Sá, R. M. P. F. D., & Gurgel, I. G. D. (2017). Healthier Sairé: a intersectorial policy as a turning point for local equity. *Ciencia & saude coletiva* , *22* , 3893-3902.	2017	Brazil	Local	Health equity	Aim to explain how intersectoriality has contributed to local equity policy within Saire municipality, including motivations for collaboration, facilitators and challenges, uncertainties and controversies for local policy actors.	Case study, incl participant observation, analysis of documents, interviews, reports of significant events, creation of a timeframe.	11 municipal managers, local authority powers and local civil society. Majority had training in health promotion or healthy municipalities.	Actor-Network Theory	No		Enabler: Participation in the Pernambuco Healthy Municipalities Network<br>Enabler: Recognised value in training e.g. healthy municipalities training<br>Enabler: Re-establishement of a Master Plan for local agendas that put into practice the commitments between management and population.<br>Enabler: Strategic leadership by promoters of Healthy Municipalities<br>Enabler: Critical events can support health equity e.g. Social Capital Assessment Survey coordinated by a University, Training of Healthy Municipality Promoters<br>Enabler: Healthier Saire intervention - brought together actors to put create concrete actions towards health equity		Nil

Hagen, S., Torp, S., Helgesen, M., & Fosse, E. (2017). Promoting health by addressing living conditions in Norwegian municipalities. *Health promotion international* , *32* (6), 977-987.	2017	Norway	Local	HiAP	Aim to determine if municipalities believe they are capable of reducing social inequalities, whether they see living conditions as a priority in local health promotion plans and to explore if structural factors, guidance and incentives from national and regional levels and local HiAP strategies are associated with designating living conditions as a main challenge or priority.	Use of a questionnaire (Schou et al 2014 – not in English) distributed to all 428 Norwegian municipalities.<br>Also used register data from Statistics Norway and Norwegian Social Science Data Services.	Mostly CEOs and public health coordinators responded.<br>75% response rate (n=361)	None	No		Enabler: Consideration of living conditions as the main challenge for local health promotion was mostly associated with use of health profiles provided by the Norwegian Public Health Institute or reporting that the municipality was capable of reducing social inequalities.<br>Enabler: Prioritising living conditions was mostly associated with having cross-sectoral working groups, inter-municipal collaborations and reporting that the municipality was capable of reducing social inequalities.	Living conditions are defined as the economic circumstances, housing, employment and education factors (Ministry of Health and Care Services).<br>82% of municipalities reported being capable of reducing social inequalities in health.<br>48% reported living conditions as their main priority.<br>Political affiliation and incentives from national and regional government had no impact on prioritisation of living conditions.	Larger municipalities were more likely to report that living conditions were a main challenge.
Helgesen, M. K., Fosse, E., & Hagen, S. (2017). Capacity to reduce inequities in health in Norwegian municipalities. *Scandinavian journal of public health* , *45* (18_suppl), 77-82.	2017	Norway	Local	Health inequities	Aim to determine factors that influence capacity of municipalities to address health inequities.	Method: Synthesis of articles and reports using the survey data in 2011 and 2014.	CEO's, civil servants<br>Survey 2011: 361 municipalities<br>Survey 2014: 325 municipalities	None	No		Pre-requisites for public health capacity were identified as :<br>Financial resources – The national level of government and county municipalities determine the public health priorities for municipalities. Any locally identified priorities have to be funded within municipality budgets (barrier). Municipalities that have done some public health planning are most advantaged in accessing these funds from higher tiers of government.<br>Partnerships – vertical governance structures rely on municipalities partnering with county municipalities in a project (incentive of funding).<br>Horizontal cooperation within a municipality: 62% of municipalities have intersectoral working groups related to public health (enabler). 41% of municipalities have planning, environmental or technical department involved in these working groups.<br>Organisational structure – as above 62% of municipalities have intersectoral working groups. Those that employ a Public Health Coordinator – most are positioned within the health sector which can be difficult to work with other sectors, 27% are employed in the Chief Executive office, which is more cross-cutting across policies, 7% in technical department which includes planning.<br>Workforce and competence: Employing a PHC has increased from 76% in 2011 to 85% of municipalities in 2014. Preference for these to be employed in positions with some power.<br>Development of health overview reports increased from 18% to 38% of municipalities between 2011-2014.	Within municipality control regarding capacity to address health inequities is the ability to employ a Public Health Coordinator and establish intersectoral working groups. PHC's would preferably have skills to develop local health profiles.	Nil
Holt, D. H., Frohlich, K. L., Tjørnhøj-Thomsen, T., & Clavier, C. (2017). Intersectoriality in Danish municipalities: corrupting the social determinants of health?. *Health Promotion International* , *32* (5), 881-890.	2017	Denmark	Local	Determinants of health	Aim to explore the role of intersectoral policy process in addressing determinants of health as an intervention strategy.	Fieldwork: 10 municipalities of varies size, socio-economic situation, location/region and political and administrative organisation of public health.<br>49 interviews + informal interviews with civil servants.<br>Participant observation, of meetings and informal conversations.	Civil servants	Theory of organisational neo-instituionalism	No		Barrier: Difficulties in engaging with other sectors;<br>Barrier: Health is a 'means to achieve other objectives' (pg 5)<br>Barrier: Health was seen as a separate issue to social issues – therefore the social issues were not considered "part of intersectoral policymaking for health". This resulted in health interventions being more behavioural focussed.	SdoH was not a concept used by local municipalities. It was often framed around 'living conditions'.<br>SdoH was seen as a reason for intersectoral action.<br>Addressing SdoH does not require anything explicitly related to the concept of health. However, if not framed as health then this aspect of the policy intent is lost, or at least the legitimacy of the involvement for health reasons is lost.<br>Findings:<br>Non-health sectors do not need to explicitly address 'health' as their problem.<br>Health should be re-framed to 'strike a balance' between health/social/economic policy objectives.	Nil

Lawless, A., Lane, A., Lewis, F. A., Baum, F., & Harris, P. (2017). Social determinants of health and local government: understanding and uptake of ideas in two Australian states. Australian and New Zealand journal of public health, 41(2), 204-209.	2017	Australia	Local	SDoH	To examine the awareness and perceptions of LG staff regarding SDoH and health equity	Online survey to 135 staff at 20 councils in SA and NSW (randomly selected)	96 LG staff with public health responsibilities across 17 councils.	None	No		Barrier: The different language used for 'public health' may be a barrier to collaborations across sectors, respondents in SA more likely to agree that health promotion responsibility at LG level is due to 'inappropriate health sector funding cuts'	11.6% of respondents had little or no familiarity with ideas about the broad determinants of health<br>35.8% rated moderately familiar with broad doh.<br>73% agreed (strongly or mildly) that sdoh knowledge influenced policy, 81% agreed they were "always trying to improve health when developing policy"<br>56.7% familiar with Ottawa charter, 52.2% with Commission on sdoh final report.<br>90% agreed that government policy and planning should include action on sdoh<br>27% "agreed that there were organisational incentives to build public health ideas into their work"<br>Felt needs by respondents - >75% more practical information on effective interventions, cross sectoral government decision making structures, new resources to gather info and evidence, health impact assessments.<br>47% strongly or mildly agreed that lifesylte choices affected people's health more than other factors.<br>57% strongly or mildly disagreed that healthcare system could reduce gap between Indig/non-indig Australians.<br>Use of evidence – all sources were seen as important including discussions with colleagues and professional contacts (93.3%), government or professional reports (89.9%), professional conferences, meetings (83.1%), research articles or books (83%), local gov resources (74.2%) and media (70.8%).<br>EHO's reported having little influence and power to influence policy.<br>EHO's reported no feedback loop to influence state health policy.	Non-metro councils less familiar with broad Doh
Von Heimburg, D., & Hakkebo, B. (2017). Health and equity in all policies in local government: processes and outcomes in two Norwegian municipalities. Scandinavian journal of public health, 45(18_suppl), 68-76.	2017	Norway	Local	HiAP	Identify enabling factors to the implementation of HiAP in LG policy (Health and equity in all policies).	Case study (x2) time series account	Strategic municipal staff (also the authors)	MSF	Yes	Authors reference MSF in discussion briefly.	There are 2 key success factors:<br>*System capacity:*<br>Previously, leaders did not refer to policies as they didn't know they existed and they didn't get implemented. These staff put in place a governance system tool, based on 'societal planning' that interconnected the different municipal plans and culminated in the Municipal Master Plan. A learning was the need to engage stakeholders and 'win their hearts and minds'. Ongoing co-creation of goals is built on trust between different policy actors. Evidence used often was through citizen voices and sharing of their stories of disadvantage.<br>*Human capacity:*<br>Municipal leaders established a strategic development unit (before the Public Health Act was in place) that focused on a range of professions, including health. The strategic unit has the role of working on governance systems and building connections between stakeholders and across departments.	The authors are strategic development staff within the municipalities who proposed public health as integrated into municipal master planning, rather than having a separate plan.<br>Used data that were analysed to represent social gradients and research on social inequalities in health.<br>Also at a time where the national Public Health Act (2012) was passed, which focused on the HiAP approach. This was considered important to keep the plan focused on equity.<br>These 'change agents' developed a 'framing document', which created some mutual understanding of health, using a salutogenic and human rights focus. The proposal focused on 'whole of government', 'whole of society', 'determinants of health' and 'ecological' perspectives, which also aligned to the WHO Health 2020 agenda.<br>The 'framing document' was brought up consistently with politicians and other stakeholders at the municipal level. Through conversations with stakeholders, created new shared knowledge of different perspectives across many sectors.<br>The Municipal Master Plan was adapted to focus on its interconnection between all plans and policies, with health and wellbeing at the core of societal development.<br>The staff came up with a range of indicators, aligned with annual reporting processes and access to local data (of which there was a large public health study making this available at the time). The data were disaggregated for geography, age, sex and social status.<br>The case study sites became members of the WHO Healthy Cities Norwegian Network – to help support them with their implementation process.	Nil
Fisher, M. (2018). Challenging Institutional Norms to Improve Local-Level Policy for Health and Health Equity: Comment on" Health Promotion at Local Level in Norway: The Use of Public Health Coordinators and Health Overviews to Promote Fair Distribution Among Social Groups". International journal of health policy and management, 7(10), 968.	2018	n/a	n/a	health equity	Response to Hagen et al 2018, discussing the idea of place-based policy action for public health, as opposed to the niter-sectoral, whole of government approach taken in Norway by Hagen and colleagues.	Commentary	n/a	None	No		Argues that 'interventions' for health action are implemented because they remain comfortable for all of the stakeholders involved. Approaches that empower and involve communities are likely to be uncomfortable.<br>Fisher proposes 3 reasons they might be uncomfortable:<br>1) Organisations are not in control<br>2) no tangible 'delivery' and about a longer term 'process'<br>3) requires organisations to view people as capable, not merely passive consumers.	Government could maybe learn from research on community-based social development and empowerment.	Nil

Fosse, E., Helgesen, M. K., Hagen, S., & Torp, S. (2018). Addressing the social determinants of health at the local level: Opportunities and challenges. *Scandinavian journal of public health* , *46* (20_suppl), 47-52.	2018	Norway	Local & National	HiAP	Aim to answer 1) how municipalities could contribute to reduce social inequalities and level the social gradient and 2)How can municipalities contribute to develop intersectoral responsibility and achieve a Health in All Policies approach to public health?	Method: Synthesis of information from 5 other articles, in addition some reports. (all five studies are already included in this lit review).	National policy-makers (interviews), municipal CEOs (survey), municipality policymakers (interviews)	None	No			Of the studies included - conclude that municipalities are responding to the national public health act to address health inequities, though most focused on behavioural and healthcare actions and few described a gradient approach.<br>A public health coordinator is identified as a 'intersectoral facilitator', although of those that employed a PHC, only 22% were employed full time. This might limit the impact these coordinators can have.<br>Health overviews were not used to guide the policy process by many municipalities - used to prioritise action program (12%) or the Master Plan (4%).	Nil
Hagen, S., Øvergård, K. I., Helgesen, M., Fosse, E., & Torp, S. (2018). Health promotion at local level in Norway: the use of public health coordinators and health overviews to promote fair distribution among social groups. *International journal of health policy and management* , *7* (9), 807.	2018	Norway	Local	HiAP	Aim to understand whether the use of public health coordinators has changed since the introduction of the public health act (2012), to what extent municipalities prioritise health inequities/social justice and whether this changes their health overviews.	Questionnaires used from 2 previous studies in 2011 and 2014 (considered pre and post the PHA 2012).<br>All 428 municipalities were included.	Not stated	None	No		Enabler: Having a health overview, either developed before or after the PHA, was positively associated with prioritising fair distribution among social groups in political decision making (as opposed to never having developed a health overview).	Between 2011-2014: An additional 16% of municipalities employed a PHC after the new Public health act (a majority of 70% already had a PHC)<br>Between 2011-2014, An additional 30% of municipalities developed a health overview (only 12% had one prior bringing the total to 42%).	Larger municipalities and less central municipalities were more likely to prioritise fair distribution in municipal decision making.
Holt, D. H., Carey, G., & Rod, M. H. (2018). Time to dismiss the idea of a structural fix within government? An analysis of intersectoral action for health in Danish municipalities. *Scandinavian journal of public health* , *46* (22_suppl), 48-57.	2018	Denmark	Local	HiAP	Aim to examine the role of organisational structure on intersectoral action for health.	Interviews with civil servants in 10 municipalities	49 interviews with Health and non-health civil servants (top and mid-level bureaucracy) across 10 municipalities (of varying size, location, SES etc).	None	No		Enabler: Leadership at all levels of bureaucracy was reported as vital for policy decisions to be put into practice.	All 10 municipalities reported reorganisations within the organisation to attempt to find a structure that worked for intersectoral action for public health.<br>The study found no ideal structure, with any organisational structure having advantages and disadvantages.<br>The two most common examples of structures were 'central unit' and 'intersectoral committee'.<br>Public health staff in central units found their central unit agenda to support other departments overshadowed their public health agenda. This did not achieve any heightened visibility of public health in the organisation and actually decreased the teams ability to collaborate with other departments. This is because public health staff were used to delivering services with other local departments and the reorganisation of their role to be more strategic conflicted with this. The staff did not have the skills in a strategic role to adapt.<br>The municipalities that had intersectoral committees found mixed levels of success. Some did not function well. Health was still not found to be a priority of other non-health sectors Respondents generally reported a lack of commitment and priority across bureaucratic departments	Nil
Holt, D. H., Rod, M. H., Waldorff, S. B., & Tjørnhøj-Thomsen, T. (2018). Elusive implementation: an ethnographic study of intersectoral policymaking for health. BMC health services research, 18(1), 1-12.	2018	Denmark	Local	SDoH	Aim to describe the process from adoption of a local health policy to implementation.	Single case study. Use of ethnographic methodology. Field notes from participation in meetings and interviews.	Municipality senior management and staff, various steering committees.	Organizational neo-institutionalism	No		Barrier: Having too many objectives and priorities made planning specific activities too difficult.<br>Barrier: Staff involved in writing the strategy wanted specific and easily communicated actions. Top management found them too explicit and did not want to commit. Therefore, actions in the implementation strategy became quite vague. This also resulted in less clear responsibilities for each department as the plan was quite abstract.<br>Barrier: The strategy couldn't have specific activities, as the council would not approve the budget that would be required to implement them.<br>Barrier: Conflicts over who should make decisions was implicitly raised. Staff wanted greater buy in and political leadership around decisions of what should be in the plan, but also did not want politicians to interfere with the details.<br>Enabler: Even though the strategy was abstract, it kept the possibility for action on the agenda and kept intersectoral action as a priority.	Intersectoral action was a high priority for the municipality (although this was never defined). Authors state that this reflects the notion of 'rationalised myth', in that it appears to be the right thing to do. The perception was also that working intersectorally would be cost-efficient. Staff and politicians had good intentions, this was 'decoupled' to the vague actions proposed.	Nil

Holt, D. H., Waldorff, S. B., Tjørnhøj-Thomsen, T., & Rod, M. H. (2018). Ambiguous expectations for intersectoral action for health: a document analysis of the Danish case. Critical Public Health, 28(1), 35-47.	2018	Denmark	Local	Determinants of health	To determine how intersectoral action is portrayed and utilised in municipality policy.	Document analysis	10 documents - based on 2 national level actors that represent Danish municipalities.	Organisational neo-institutionalism	No			The terminology used for intersectoral action for health were mostly elusive and vague e.g. thinking health, creating coherence.<br>The ideal of intersectoral action was evident, though not specific actions to achieve this, a notion referred to as a rationalised myth.<br>The rationale or argument for intersectoral action for health outcomes was based on efficiencies, economic incentives and improved quality of services.<br>High level management and political support was highlighted in documents as supportive and necessary.<br>Conclude that intersectoral action for health is mostly rhetoric in policies, rather than having any practical impact on health outcomes in practice.<br>This 'decoupling' of organisational ideals with the operational reality might help explain the challenges to intersectoral action.	
Lillefjell, M., Magnus, E., Knudtsen, M. S., Wist, G., Horghagen, S., Espnes, G. A., ... & Anthun, K. S. (2018). Governance for public health and health equity: The Tröndelag model for public health work. Scandinavian journal of public health, 46(22_suppl), 37-47.	2018	Norway	Local	Health equity	Aim to understand how to strengthen the capacity of municipalities to work more systematically, knowledge-based and multi-sectoral to address health inequities, including presenting a model.	Three case study municipalities, incl. document analysis and interviews (n=30). Interviews or focus groups at 3 time points (9 at baseline, 1 at mid-point and 20 at completion). Document analysis of strategic and planning documents, meeting minutes from all 3 municipalities at baseline. Observational data of three planning	Municipal leaders of plans, policies or organisational responsiblities from different departments. Researchers also participated in informal meetings with citizens.	None	No		Enablers to a systematic, knowledge-based and multi-sectoral approach to public health requires:<br>• Political and community commitment<br>• Shared understanding of population health<br>• Cooperation across sectors and levels<br>• Anchoring public health in municipal planning<br>• Identification of relevant sources of knowledge outside of the health sector<br>• Engaging several target groups<br>• Fostering participation of citizens<br>• Opportunities for knowledge sharing through multi-sectoral governance<br>• Evaluation and feedback loop for future decision-making consideration	Based on the facilitators identified, authors proposed a model for public health work in municipalities which included 7 steps, each to be completed before moving on to the next step:<br>Societal mission: ('anchor' point such as Norwegian Public Health Act)<br>Defining the knowledge base: (from different evidence sources and establishing a common understanding)<br>Involving and developing: concrete plan for strategy intervention, involving citizens in planning.<br>Planning new initiatives: delegating responsibilities to build local ownership.<br>Implementing: processes documented, to allow for adjustments based on budget, political situation.<br>Evaluating: Evaluation plan to be done when planning interventions, collaborate with researchers.<br>Turning action into new knowledge: Evaluation findings transferred to new policy and planning processes.<br>The model has not yet been tested.	Nil
McCosker, A., Matan, A., & Marinova, D. (2018). Policies, politics, and paradigms: Healthy planning in Australian local government. Sustainability, 10(4), 1008.	2018	Australia	Local	Healthy planning	Aim to identify the barriers and enablers to the update and implementation of healthy planning and active living initiatives in LG in Australia.	Qualitative semi-structure interviews	Government employees at regional level (n=6), LG (n=5) and state (n=4), also roles in academic (n=4), NGO's (n=3) and private sector (n=3).	MSF	Yes	The MSF is presented as a theoretical lens, results are presented using streams of MSF, but the discussion doesn't bring these parts back together to explain the policy process.	Enabler: State level legislation e.g. mandating LG public health plans. Although this was mixed in terms of level of support for incorporating concepts of health in planning – but beneficial as a mandate particularly where it offered funding initiatives or further resourcing.<br>Enabler: Research and guidelines were considered relevant evidence that supported healthy planning.<br>Enabler: Advocates played the role of a knowledge broker.<br>Enabler: community demand<br>Enabler: working in partnerships<br>Enabler: framing 'health' as something else that is a co-benefit. Terms such as 'liveability' and 'wellbeing' were seen as more marketable than the term 'health'. Advocacy messages needed to be different for each LG.<br>Enabler: Healthy urban planning more likely to be implemented if there was an economic or political benefit.<br>Barrier: State and local policies are not sufficient for implementation – local policy adhoc and usually required motivated staff to deliver on it.<br>Barrier: It was considered difficult to get local level data.<br>Barrier: Politicised decision making, timeframes needed for outcomes didn't match short term political elections. Decisions on healthy planning are political – reliance on elected members to decide. Came with a lack of evidence-base and more politically driven decisions based on popularity or marketability. Required policy entrepreneurs or champions to make it happen.<br>Barrier: The idea of addressing healthy urban environments and developing policy was more politically favourable than actually implementing it – not having specific strategies in the plan that might not be accepted (or be seen as controversial) by community.	Community health was seen as a co-benefit, not the reason, for action. It was a barrier if it was seen as something additional to current practice.	States with and without legislation had similar barriers.

Scheele, C. E., Little, I., & Diderichsen, F. (2018). Governing health equity in Scandinavian municipalities: The inter-sectoral challenge. *Scandinavian journal of public health* , *46* (1), 57-67.	2018	Denmark, Norway, Sweden	Local & Regional	HiAP	Aim to analyse the factors that influence implementation of HEiAP (health equity in all policies) governance.	Qualitative interviews (n=20)	Local and regional government administration and politicians. Interviewees were selected across different departments (horizontal) and different levels of management (vertical). Municipalities were included from Norway (n=3), Sweden (n=4) and Denmark (n=3) and a regional government from Sweden (n=1).	MSF	Yes	The authors claim that the results are inspired by the Multiple Streams Framework, though the framework is never used explicitly.	Barrier: Political commitment: Whilst all of the municipalities had a written commitment to HEiAP, there was not always the action to follow it through.<br>Barrier: Political level budgeting: There is a lack of evidence on the cost-effectiveness of action, making it difficult to advocate for health equity interventions.<br>Enabler: Horizontal coordination:Municipalities that framed health inequity outside the health discourse were more successful at horizontal cooperation (eg. social sustainability). However, this risks losing sight of the health issues.<br>Barrier: Vertical coordination: Regional and local level coordination is difficult as there are a lot of municipalities in each region, each unique with their own needs.<br>Enabler: Evidence: Having data that reflects health inequities is a motivator for municipalities to take action.<br>Barrier: Evidence for interventions/actions is limited.<br>Barrier: Monitoring action on health equity is difficult. There lacks a 'common language' to be able to report against.<br>Barrier: One of the key challenges to inter-sectoral policy making in local governments is the structure of local government administrations.	The authors recommend that health equity impact assessments can help maintain a focus on health across sectors.	Nil
Synnevåg, E. S., Amdam, R., & Fosse, E. (2018). Intersectoral planning for public health: dilemmas and challenges. *International journal of health policy and management* , *7* (11), 982.	2018	Norway	Local	HiAP	Aim to describe the experience of planning as a tool to implement HiAP in Norwegian municipalities.	Case study (n=3) of different size (geographical and population).	30 interviews with employees and politicians (included CEOs, mayors, politicians, public health coordinators, managers of other departments, planners).	Davoudi's four properties of planning	No		Enabler: Balance between quantitative evidence and the qualitative discussions.<br>Enabler: Need for top down support (e.g. goals in master plans), but also support from operational staff as they need to be able to implement the action. It is acknowledged that sometimes this is difficult.<br>Barrier: Putting public health first might be met with 'distrust' by other departments and be counterproductive to action on HiAP. Respect and power must be shared across sectors to find common ground.<br>Barrier: Structures of municipalities do not always promote intersectoral participation e.g. vertical communication between operational and executive level staff.	There is tension between the need for instrumental, structural planning processes, such as having clear goals included in higher level planning documents or routine HIA's or similar procedures, with the need for more process-oriented procedures that involved discussions and dialogue between departments.<br>Debate was whether to frame the planning action as public health and have a separate public health plan, or whether to integrate public health into the master plan. There were pros and cons seen for both approaches. Having a specific public health plan helps to bring attention to the issue, although can be seen as public health being 'special' or have some extra power (and be experienced as threatening to some). A more integrated approach that is aligned to already existing work is the alternative.<br>Authors debate the challenge of moving from 'cross sectoral' to 'intersectoral' planning (which they define as more interdisiplinary action).<br>Agree with other researchers that 'health imperialism' is a barrier to HiAP.	Nil
Synnevåg, E. S., Amdam, R., & Fosse, E. (2018). Public health terminology: Hindrance to a Health in All Policies approach?. *Scandinavian Journal of Public Health* , *46* (1), 68-73.	2018	Norway	Local	HiAP	The aim of the study is to determine if the terms 'public health' and 'public health work' are suitable and transferable terms for municipalities in implementing a HiAP approach.	Case study design (n=3), incl. semi-structured interviews and document analysis.	30 interviews with those been involved in public health planning (included CEO's, Mayors, department leaders).	Institutional translational theory	No		Enabler: Reframing the term 'public health', without taking away the intent of what the national plans mandated e.g. Case study site 3 used the term 'living conditions'. This term was seen as more relevant to the local context.<br>? : Some felt that the term 'public health' needed to be used initially so that staff in different departments understood their role and potential impact on health, however the terms then became irrelevant in actual practice.<br>Barrier: The terms 'public health' and 'public health work' were perceived as complex terms that were difficult to define what they meant.	The authors conclude that the terms can be a hindrance to addressing their intent to address HiAP. The terms may be needed initially to understand the reason for considering health impacts, though at some point in planning the terms could be re-framed.	Nil
Van Vliet, J. (2018). How to apply the evidence-based recommendations for greater health equity into policymaking and action at the local level?. *Scandinavian journal of public health* , *46* (22_suppl), 28-36.	2018	Sweden	Local	Health inequities	Aim to reflect on the process of local municipalities implementing regional health policy recommendations.	Discussion: personal reflection and experience of author.	None	None	No			The politicians from the 13 local municipalities in the region came together and agreed on 10 priorities of action.<br>Documents in the case study site (municipality) demonstrated that the priority areas were incorporated into local budget plans (hence prioritised in the budget). These were established during the regional commission process, drawing on the evidence-based recommendations.<br>Norrkoping was already investing in social service and cross sectoral interventions prior to the regional health equity commission efforts (e.g. education, childcare). This included significant financial investment in social services.<br>It is acknowledged by the author that local municipalities could not just add more priorities to their list, though needed to integrate the recommendations into existing political priorities. This allowed for each local municipality to make the adjustments for their local region needs.<br>In the instance of Norrkoping, many of the 10 agreed priority areas were already invested in. The author was able to map these to existing investments. It is acknowledged that advocating for this to happen may result in a more 'tick and flick' exercise.<br>Action on health equity risks just targeting the most disadvantaged, rather than distributed across level of advantage.	Nil

Browne, G. R., Davern, M., & Giles-Corti, B. (2018). 'Punching above their weight': a qualitative examination of local governments' organisational efficacy to improve the social determinants of health. Australian and New Zealand Journal of Public Health.	2019	Australia	Local	SDoH	Aim to describe how LG in Victoria conceptualised their organisational efficacy to address public health with reference to their statutory obligations.<br><br>Answered to what extent LG consider the State priorities when developing MPHP & how effective they feel they can address SDoH.	Qual interviews	16 interviews with health planners (background in HP, community development, EH). These staff are responsible for developing the MPHP (so some bias). Across rural, regional and city areas.	None	No		Enabler: LG planners had good understanding of socio-ecological model of health and demonstrated upstream strategies that impact HWB were considered. Good recall and use of the 'Environments for Health' resource made available to LG (better than talking about determinants of health).<br>Enabler: Interviewees saw a responsibility for community wellbeing and saw upstream initiatives as important. In fact, across the 4 key action areas, addressing social determinants was the top priority for health (2nd – health protections and 3rd – behavioural program and 4th – preventive healthcare). However, noted that behavioural programs (eg. Walking groups) were 'politically attractive'.<br>Enabler: LG see 'upstream' investments more cost effective than one-off behavioural programs.<br>Barrier: Victorian LG do not get funding to implement public health plans – so planners consider what is feasible and within the realm of LG. Do not duplicate what other sectors are already doing.<br>Barrier: Community consultation with community members was perceived as reverting to behavioural programs as suggestions for HWB, rather than thinking upstream – which perpetuates the cycle of what is the local priority.	LG adopted socio-ecological model of health. The state priorities were considered but less influential than considering local needs. Planners felt that the State priorities were missing key social factors eg. Family violence, gambling etc.<br>Respondents took the view that "everything LG does plays some role in creating HWB".<br>Finding: There needs to be more collaboration between state and local government to identify health priorities.	Did not consider results based on rurality/geographical location although the sample included a mix of these.
Fosse, E., Sherriff, N., & Helgesen, M. (2019). Leveling the Social Gradient in Health at the Local Level: Applying the Gradient Equity Lens to Norwegian Local Public Health Policy. *International Journal of Health Services* , *49* (3), 538-554.	2019	Norway	Local	HiAP	Aim to describe how local governments have implemented the national Norwegian Public Health Act 2021.	Uses 2 other primary data sources including survey data from 2011, 2014 and 2017, along with qualitative interviews from 6 municipality case study sites.	CEO's, policymakers	Gradient evaluatio n framewor k	No			The proportion of municipalities that completed a health overview report increased from 39% in 2014 to 85% in 2017.<br>Awareness of the national public health act was high amongst public health coordinators in municipalities.<br>In 2017, policy areas covering social determinants of health (such as housing, education, income) had a low priority. 36% of municipalities gave priority to these areas, as opposed to 58% prioritised individual mental health issues amongst children (which was also a nationally funded program at the time which could influence this prioritisation).<br>Few interviewees were aware of the social gradient of health.<br>Between 2014 – 2017 more municipalities had established intersectoral working groups, from 62% to 72%. Between 2011 to 2014, CEO staff and planning department staff increased in participation on intersectoral working groups (CEO up from 22% to 68%; Planning staff up from 16% to 65%), though this dropped in 2017 (56% for both).<br>Municipalities are increasingly reporting capability in addressing health inequities, up from 83% in 2014 to 95% in 2017.<br>There seems to be a good understanding of the investment in the early years to address health inequities later in life, such as investing in kindergartens and childcare.<br>Most municipalities still prioritise individual lifestyle issues, 34% in 2011 and 71% in 2014. In 2017, mental health problems were prioritised most by 62%.	Larger municipalities tend to prioritise living conditions and smaller municipalities prioritise lifestyle issues.
Kneale, D., Rojas-García, A., & Thomas, J. (2019). Obstacles and opportunities to using research evidence in local public health decision-making in England. *Health research policy and systems* , *17* (1), 1-11.	2019	England	Local	Public health	Aim to explore how evidence is used in local public health decisions given the reorganisation of public health responsibilities to a local government context.	Qualitative Interviews	12 public health practitioners (directors, managers) within 3 local authorities.	None	No		Enablers:<br>Stable administration (able to take more risks), "jargon-free" evidence, economic evidence (highlight short term investments had long term savings), Qualitative research helpful contribution (although some thought is opened opportunities for decision-makers to inflate with anecdote).<br>Local evidence was powerful.<br><br>Barriers:<br>Majority felt that political ideologies sometimes contradicted need (as they weren't seen as favourable for political reputations or election).<br>Time pressures was a barrier to utilising evidence and timing of data released by other sources not always 'timely'.<br>National public health policies weren't always seen to translate to a local population.	Elected officials relied on anecdote, which was also recognised as important to engage with communities.<br>Evidence was not used in public documents.<br>Community input was seen as overriding evidence.<br>Conclusions: The shift of public health to LG has changed the way evidence is used and how public health practitioners' 'package' this evidence. There needs to be a balance between anecdote and evidence and a larger focus on the creation and use of local data for complex local systems.	Nil
Kokkinen, L., Muntaner, C., O'Campo, P., Freiler, A., Oneka, G., & Shankardass, K. (2019). Implementation of Health 2015 public health program in Finland: a welfare state in transition. *Health promotion international* , *34* (2), 258-268.	2019	Finland	Local & National	HiAP	Aim to examine how the Health 2015 policy was affected by changes in the role of the state.	Interviews and literature.<br>10 informant interviews along with peer review and grey literature to support claims. Interviews explored for barriers and facilitators to the implementation of Health 2015.	The informants were selected based on their knowledge of HiAP implementation across diverse sectors of national government.	None	No		Barrier: When national legislation was restructured so that municipalities have greater freedom with no financial or legislative arrangements remaining in place from the state, Health promotion investment decreased as a result, with funding cuts to health promotion, more being outsourced and therefore ad hoc in nautre.<br>Barrier: HIA, which mandatory in 2010, lacked the workforce capacity to be able to meet legislative requirements.<br>Barrier: The increasing private industry influence had greater lobbying influence on state decision makers (e.g. direct funding, greater access to decision-makers) and has resulted in a lifestyle drift towards an individualistic approach.<br>Barrier: Ideological interests in economic growth meant that public health objectives got a lower priority.		Nil

Lilly, K., Hallett, J., Robinson, S., & Selvey, L. A. (2020). Insights into local health and wellbeing policy process in Australia. Health promotion international, 35(5), 925-934.	2019	Australia	Local	HWB	To investigate factors that enable or challenge the initiation and actioning of LG health and wellbeing policy.	Researchers also participated in informal meetings with citizens.	243 CEO's, 1096 elected members, 135 strategic and 160 operational managers and 191 staff in LG.	MSF, ACF, PEF and ADEPT	Yes	Informed questionnaire design using relevant aspects from each of the four framework. Discussion describes how the findings align with factors of political science frameworks.	Enablers: Good understanding of HWB as a policy problem, Strong council committment to HWB, personal & professional obligation for HWB shared amongst decision makers, political will to act, sufficient cooperation internally, perceived cost-effectiveness.<br><br>Barriers: Funding resources, support by other sectors, level of support and leadership from higher tiers of government, lack of staff capacity, No key champions for HWB in Australian LG. Combined with lack of lobbying internally could challenge HWB reaching policy agenda.	16% of respondents reported HWB being considered in a rang eof policy areas.<br>6.5% were able to respond that 'all policies impact' to an open ended question on what policy areas are able to improve community wellbeing.<br>58% reported health broadly from a determinants of health and health equity perspective.<br>HWB given a high priority - 43% an >=8 on a scale of 10	City and rural councils reported HWB as a higher priority than regional councils.<br>City respondents more likely to report personal obligation and organisational obligation and that it is part of their professional duties.<br>City councils more likely to report a more supportive legislative environment.<br>City and regional council more likely to report strong leadership within council and sufficient cooperation during policy development.<br>City councils more likely to report funding and staff capacity.<br>Funding was a particular barrier for rural councils.
Mundo, W., Manetta, P., Fort, M. P., & Sauaia, A. (2019). A qualitative study of health in all policies at the local level. *INQUIRY: The Journal of Health Care Organization, Provision, and Financing* , *56* , 0046958019874153.	2019	USA	Local	HiAP	Aim to describe how Colorado local public health agencies are implementing HiAP, as well as successes, tools and challenges associated with the implementation.	Qualitative interviews (n=13)	8 Directors and Deputy Directors of LPHA's, 5 experts in local public health policy.	None	No		Enabler: Developing trust with external agencies through regular contact or finding shared values.<br>Barrier: Lack of understanding of the determinants of health and the role of local government to address. Also needed to educate partners outside of health.<br>Barrier: Limited staff to be able to implement HiAP. Some agencies do have a specific role for implementing HiAP.<br>Enabler: Having access to decision-makers to learn about the impacts of policy on health inequities.<br>Enabler: Directly liaising with community that were affected by policies, to generate personal stories.<br>Barrier: Lack of funding. Funding is usually earmarked for specific health priorities, making it difficult to be responsive to structural determinants. Also high staff turnover, insufficient community spaces for engagement and lack of technology and equipment.<br>Barrier: Lack of direction from the state government<br>Barrier: Lack of political will. Mostly as the framing of messages from the state and public health community was lacking, which meant HiAP did not get raised on the political agenda.<br>Enabler: Tools such as data sharing platforms, community health assessment tools (although these require staff time and expertise which may not be available in all LPHA's).	The state government needs HiAP to be a higher priority for this to filter down to local level. Colorado currently has no state mandate for HiAP.	Purposely included a range of 8 different geographical types and size of LPHA's but did not present findings to compare.
Synnevåg, E. S., Amdam, R., & Fosse, E. (2019). Legitimising Inter-Sectoral Public Health Policies: A Challenge for Professional Identities?. *International Journal of Integrated Care* , *19* (4).	2019	Norway	Local	HiAP	Aim to discuss how professional identities relates to implementation of intersectoral collaboration.	Case study design (n=3), incl. interviews .<br>Sites of differing geographical areas and population size.	31 participants involved in public health policy across 30 interviews. Incl administration positions, politicians, department leaders.	Scotts theoretical framework of institutional pillors; Suchmans<br><br>legitimacy types	No		Enabler: Regulatory nature of HiAP has supported the legitimising of the HiAP approach.<br>Enabler: There is motivation in local municipalities to develop local regulations and structures for HiAP (e.g. HIA).<br>Barrier: Lack of awareness of public health goals related to the daily work within different sectors.<br>Enabler: Many respondents, outside of public health departments, did recognise that public health was part of their responsibility. However, they report that health is not always the reason, drive or issue that is most important to them in their work.<br>Barrier: Conflicts with professional identities may inhibit the intersectoral collaboration required to achieve HiAP.<br><br>Barrier: Perception that staff outside public health were 'being told what to do', without consideration of actions already being done (e.g. from a cultural perspective).	*Regulative legitimacy* (laws) was apparent in all 3 municipalities. The national public health act legislates HiAP and locally there are regulations and structures in place e.g. integration into local public health policies, health impact assessments.<br><br>*Normative legitimacy* (values, norms) was mixed. At both political and administrative levels, respondents reported public health as an 'issue of interest' and important. There were still doubts if politicians and/or administrative employees really understood the integration of public health.<br><br>*Cognitive legitimacy* – (understanding and knowledge) – In one municipality, understanding was clear and shared amongst informants, though most commonly referred to as 'living conditions'. In one municipality, the understanding of the HiAP approach was less clear, with no apparent understanding of social inequalities in health.<br><br>*Pragmatic legitimacy* – (perceived usefulness of the approach) – All municipalities report HiAP as useful – particularly to the community. It is less useful to their own internal department of day to day work.<br><br>Conclusion: Regulative legitimacy is less challenging to achieve than cognitive and normative legitimacy.	Case study site 2 is reported to struggle to legitimise HiAP more than the others, but no reasons to explain why this might be the case.

South, E., & Lorenc, T. (2020). Use and value of systematic reviews in English local authority public health: a qualitative study. *BMC Public Health* , *20* (1), 1-11.	2020	England	Local	Public health	Aim to determine the use of systematic reviews as evidence in policy making amongst public health directors/staff in local authorities.	Semi-structured qualitative interviews	14 Directors of Public Health or public health trained consultants (across 10 LA's)	None	No		Barriers to using systematic reviews:<br>- lack of availability,<br>- limited focus (particularly on determinants of health)<br>- elected politicians might value other sources of evidence (e.g. anecdotal evidence)<br>- lack of training in critiquing quality of evidence by other staff in LA's<br>- lack of time.	Interviewees identified that they used systematic reviews and valued the evidence from this source.<br>They also used other sources of information, particularly reports from trusted institutions e.g. NICE.	Nil
Baldwin, L., Dallaston, E., Bennett, B., McDonald, F., & Fleming, M. L. (2021). Health in all policies for rural and remote health: A role for Australian local governments?. Australian Journal of Public Administration.	2021	Australia	Local	HiAP	To discuss possible challenges to a HiAP approach for rural and remote LGs	Controversy paper	None	None	No		Barriers (these are opinions/proposed): Rural and remote LG's may have less capacity for policy development due to greater responsibilities and more time travelling, less access to public health experts to collaborate with.	Authors recommend that legislation be enacted for LG to address health and wellbeing where this doesn't already exist (although not supported by literature).<br>Authors recommend that LG in rural and remote areas require more support by higher tiers of government, including expertise and funding (although not supported by literature).	Barriers and suggestions for rural and remote LG's in Australia to adopt HiAP.
van der Graaf, P., Cheetham, M., Redgate, S., Humble, C., & Adamson, A. (2021). Co-production in local government: process, codification and capacity building of new knowledge in collective reflection spaces. Workshops findings from a UK mixed methods study. Health research policy and systems, 19(1), 1-13.	2021	UK	Local	Population health	Aim to explore perspectives from LG staff in co-developing, using and applying evidence in the commissioning of public health services.	Mixed method research.<br>This paper reports on findings from workshops (n=54).	Included representatives consisting of LG staff stakeholders e.g. universities, voluntary organisation.	Created their own model of co-production of research evidence in LG.	No		Barriers to use of evidence:<br>- Data needed at a local level did not match data available, or data required to be collected for national government.<br>- Data was collected by different departments or organisations and not shared, difficult to compare, not available at a geographical level.<br>- Priority given in one case study site for quantitative data, unsure how to use qualititative data from community.<br>- Lack of value for evaluating programs for impact, rather than just whether they were delivered or not.<br>- differing cultures between LG and academia. LG averse to negative evaluation findings and that academic research takes too long. Academics prioritising peer review publications and funding grants.<br>- Lack of shared, collaborative space for reflection on evidence aross LG departments.<br>- Departments are siloed in LG, each with their own culture.<br>- Researchers lacking awareness of LG policy processes.<br>Enabler for evidence use:<br>- Academics having trusted contacts in LG		Nil
