# Supplementary material for: Factors of the policy process influencing Health in All Policies in local government: A scoping review
Source: Front Public Health. 2023 Feb 9;11:1010335. doi: 10.3389/fpubh.2023.1010335 (PMC9949293; doi:10.3389/fpubh.2023.1010335)
Supplement: Supplementary file 2 [file Data_Sheet_2.pdf]

The policy process factors influencing HiAP in LG: A scoping review

Authors

Mrs Kara Lilly[1,2], Dr Bridie Kean[1], Prof Suzanne Robinson[2], Dr Jonathan Hallett[2], Assoc Prof Linda A. Selvey[3].

1. University of the Sunshine Coast, 2. Curtin University, 3. University of Queensland

Abstract

Objective: This scoping review aims to identify what factors of the policy processes enable and/or challenge Health in All Policies to gain political traction as an approach within local government. The review will consider different contexts of local government, and the application of theories of the policy process.

Introduction: Health in all policies is an approach to population health that requires sectors outside of health to consider their potential impacts on health outcomes. Local government has been proposed as a feasible tier of government to establish this approach, though the research on how and why is lacking. Describing this policy process could best be done through the lens of political science theories of the policy process, an underutilised approach in health promotion.

Inclusion criteria: The scoping review will include studies published in academic peer-review journal articles and thesis dissertations, along with grey literature such as government reports. Sources will be included if they refer to Health in All Policies (or a related concept), describe any aspect of the policy process and focussed within the local government setting.

Methods: Searches will be conducted in Scopus, Proquest and EBSCO for sources between 2001-2020 and in English. Two reviewers will independently review all sources for inclusion, initially based on title and abstract, followed by full text review and a manual scan of reference lists. Data extraction will be piloted by two reviewers and completed in full by one researcher. Data will be presented diagrammatically, along with a narrative summary.

Introduction

The health and wellbeing of populations is influenced by a range of social, environmental, political, economic and physical factors (Marmot et al., 2008). Addressing these influences requires the cooperation of sectors outside of the traditional healthcare model to achieve greater population health outcomes (WHO and the Government of South Australia, 2010). There have been several attempts to frame this cross-sectoral approach to population health, more recently referred to as the 'Health in All Policies' (HiAP) approach. HiAP is defined as "*an approach to public policies across sectors that systematically takes into account the health implications of decisions, seeks synergies and avoids harmful health impacts in order to improve population health and health equity*"(World Health Organization, 2014, p. i20).

Achieving a HiAP approach in practice has been challenging, as it requires a systematic approach to overcome siloed governance structures, political will and leadership (Ollila et al., 2013) and to manage the resistance from sectors outside of health to take responsibility for population health outcomes (Koivusalo, 2010). Most research to date has focussed on the national and state/provincial levels of government (Shankardass et al., 2012). It has been argued the third tier of government, termed local government (LG) or municipality, is better positioned as a more feasible tier of government to address the range of determinants of health (Burris et al., 2007; Collins & Hayes, 2013; Harris & Wills, 1997). This notion of feasibility is based on, though not limited to, the opportunity of LG to design urban neighbourhoods, influence transport and employment conditions, contribute to social capital and provide access to local facilities, whilst also being in a position to bring different sectors together to address local community needs (World Health Organization, 2012).

However, the research on how or why HiAP would be adopted by decision makers, within any tier of government is very limited (Shankardass et al., 2014). Understanding the policy process of how or why could be better described through the use of political science theory, of which researchers in health promotion conclude there has been little adoption (Breton & De Leeuw, 2011). Furthermore, in instances where political science is described, it tends to be superficial (Shankardass et al., 2012).

Therefore, this scoping review will explore what factors of the policy processes enable and/or challenge HiAP to gain political traction within the local government (LG) policy subsystem. The review will consider different LG contexts (e.g., legislative environment, geographical size and location), and the use of theories of the policy process applied to the research methodology and findings. The purpose of the scoping review is to determine to what extent these concepts have been applied together in the international body of research. As acknowledged by Peters et al. (2015), scoping reviews are appropriate for addressing research questions that go beyond a specific intervention or specific questions, which is the case in this research.

Two scoping reviews with some similarities to this topic were identified in an initial search of the databases Scopus and Proquest. Guglielmin et al. (2017) aimed to identify the barriers and enablers to HiAP at a local level. The focus of the scoping review was to identify factors that impacted the implementation of HiAP or in response to a mandated action to act on HiAP. For this reason, the review included articles within LG that had already applied or implemented HiAP, or a similar concept that aims to achieve health and wellbeing or address health inequities. Articles were excluded if they were based at a state or national tier of government, though included if LG was involved. The review also excluded articles on health impact assessment tools, unless directly linked to the HiAP process. The review included only articles written in English between the years 2002 to 2016. The authors identified 23 peer review articles and four government documents to be included.

Van Vliet-Brown et al. (2017) aimed to identify how HiAP is utilized in municipalities and identify research gaps. The scoping review reported on both recommendations for successful implementation of HiAP, though also the common themes that arose in the literature related to the barriers and enablers for adoption of the approach in LG. Articles were included from both peer review sources and grey literature between 2006 to 2015, using ‘HiAP’ specifically as a term, focussed on the local or municipal tier of government. Related concepts and other similar terms, such as health equity, were not included in the search. Articles were excluded if they referred to other tiers of government or in a language other than English. Other inclusion and exclusion criteria

were not provided. The authors identified 27 sources, including peer review research, commentaries, policy documents, government information sources and conference abstracts, posters and presentations.

The proposed scoping review in this protocol has some similarities and differences to the two reviews outlined above. The interpretation of HiAP is used similarly to Guglielmin et al. (2017), in that it uses the term 'health in all policies' both explicitly and implicitly alongside terms such as 'health equity'. This extension of the definition and understanding of the term is adopted to include it's underlying purpose of addressing a range of health determinants across different policy areas that impact populations to address health inequities (Ollila et al., 2013). This is also a key difference to the review by Van Vliet-Brown et al. (2017) which limited search terms to those relevant to the explicit use of 'health in all policies'. The role of LG, alongside other tiers of government, will also be used as per Guglielmin et al. (2017), with the addition of sources where LG has a clear role amongst other tiers of government. This is something that was also excluded in the review by (Van Vliet-Brown et al., 2017). The proposed review will also similarly exclude articles related to health impact assessment tools as per Guglielmin et al. (2017), though will consider them if they are clearly a factor in the policy process. As per both scoping reviews, sources will be in only the English language.

Overall, the proposed scoping review takes a broader perspective of the policy process. The aforementioned scoping reviews focussed only on implementation of HiAP. This scoping review will explicitly focus on the full policy process from agenda setting through to monitoring and evaluation. To guide decision-making, a deconstruction of variables related to factors in the policy process (according to various political science frameworks) has been provided to all reviewers (Appendix IV). Additionally, Guglielmin et al. (2017) focussed on studies where HiAP was already mandated, whereas the proposed scoping review will not limit to only those cases where HiAP is already legislated. It was not clear if Van Vliet-Brown et al. (2017) excluded any studies based on context. This scoping review will distinguish the research findings based on LG contexts of various geographical location and size, of which neither of the existing scoping reviews has included. In addition, the proposed scoping review will consider the application of political science theory to inform the research of the policy process, of which neither of the existing scoping reviews has applied.

In summary, the proposed scoping review provides a more in-depth and nuanced focus on the HiAP approach, locating this within the LG context, though not excluding their relationship with higher tiers of government. The scoping review will include the most recently available research (up to year 2020) and captures a broad scope to include underlying concepts of HiAP, including determinants of health and health inequities. The review includes all factors related to the policy process, not only including those LG that have already applied HiAP, through investigating factors that influence policy agenda setting, development and implementation of the HiAP approach, as well as the factors that are enabling and challenging the sustainability and evaluation of this approach. Furthermore, this scoping review takes a specific interest in the different contexts of LG and the role of political science as a theoretical lens in explaining the policy process.

The objective of this scoping review is to establish an understanding of what is currently known of how and why LG adopt (or do not adopt) a HiAP approach, including how and why HiAP gets on to the policy agenda (or why it does not) and what the barriers and enablers are to the initiation, implementation or evaluation of the approach, including across different LG contexts and

jurisdictions. The aim is to also identify the extent to which the literature has utilised theories from political science to explore and explain the policy process.

Review question

The research question proposed for this review is:

"What are the critical factors in the policy process that enable and/or challenge local government in initiating, implementing or evaluating a HiAP approach to achieve population health and wellbeing outcomes?"

Some additional sub-questions of interest include:

- *Are these identified policy factors different across various local government contexts and jurisdictions?*
- *How does the literature related to HiAP approaches in LG apply theories from political science?*

Keywords

Policy process, local health policy, health in all policies, determinants of health, healthy public policy

Eligibility criteria

Participants

For the purposes of this research, the participants are not a defined part of the population, and so this aspect of the PCC framework becomes less relevant. However, the focus of HiAP as a concept is framed on population health and wellbeing, as opposed to individual health services or health outcomes.

Concept

The concept of HiAP represents a policy process that is underpinned by addressing structural determinants of health and addressing health inequity across a range of policy areas. The term 'HiAP' is a relatively new term in the last decade, so the underlying intent of the approach will be taken into account in the inclusion of sources, including if there is reference to policy processes related to structural determinants of health (political, social, environmental (built or natural)) or concepts such as health equity. These are concepts that impact on population health and wellbeing, rather than healthcare treatment services or practices, or individual behavioural approaches to health and wellbeing. For this reason, sources will be excluded if they define health related to a biomedical or healthcare approach/setting (e.g., delivery of healthcare services), or individual behavioural approaches (e.g., physical activity).

This includes the exclusion of sources related to urban planning or the built environment that focus on specific behavioural approaches (e.g., physical activity) or disease (e.g., obesity). It is recognised that the research on urban and neighbourhood planning is likely more advanced than that of other determinants. Built or natural environment research that focus on technical aspects of zoning and

urban planning or make recommendations on what urban planners should do to improve health and wellbeing outcomes will be considered out of scope. Research related to a relevant determinant of health (e.g., built environment, housing, social strategies) will be included where it relates to population health and health inequities, and is clear that the study is related to how/why health is incorporated into policy decisions at a local level.

Sources where the definition of ‘population health’ or ‘health determinants’ is not clearly defined (e.g. the term used is ‘public health’) will be included in the review if it is apparent the focus of the research is on population health, not health care.

Given the goal of the research is to understand the policy process that makes HiAP feasible or not, all sources to be included in the review need to address at least one of the key factors that relate to the policy process (Appendix IV). This includes both explicit use of theories of the policy process (e.g., Multiple Streams Framework) or in the absence of a specific theory. For this reason, sources will be excluded if the focus is solely on policy analysis (e.g., discussing content within policies), policy impact or policy outcomes (e.g., what resulted due to policy), in the absence of understanding the processes that led to these decisions being made.

Context

The context for this research is the LG policy sub-system. The sub-system is focussed within the structures of LG, including both organisational systems and the individual decision makers. However, the policy actors can extend to other local stakeholders that influence the policy sub-system such as community-based organisations and civil society. The role of senior tiers of government will be considered where they include or relate explicitly to the influence on LG. Studies that include multiple tiers of government will be included where a clear role for LG is evident.

Given the variability in LG systems across the globe, studies within middle-high income countries will be included. Consideration will be given for studies in low-income countries where the reviewers agree there is value in their inclusion.

Types of Sources

All types of empirical studies will be considered in the review. The scoping review will include studies published in academic peer-review journal articles and thesis dissertations, along with grey literature such as government reports. It is considered appropriate to include government reports and thesis dissertations, as the concept of HiAP is relatively new and the academic literature may not capture all of the information that best informs this research topic. Opinion papers, such as commentaries and debates, will be considered in the review. Literature reviews, newspaper reports and conference abstracts will be excluded from the review.

Due to language and resource constraints, only sources written in English language will be included. Articles published between 2001 and 2020 will be deemed eligible for inclusion, reflecting 20 years of possible practice in the area of LG health and wellbeing policy. is considered necessary as the policy process can be a long-term change process and research may extend beyond several years prior to publication.

Methods

The proposed scoping review will be conducted in accordance with the JBI methodology for scoping reviews (Peters MDJ, 2020).

Search strategy

The proposed database search terms have been chosen based on the PCC framework (Peters MDJ, 2020). An initial limited search, conducted in collaboration with a librarian and several of the authors, was undertaken across several databases (Scopus, Proquest, Web of Science, EBSCO), using key search terms such as 'health in all policies', 'healthy public policy', 'local government' and 'municipality'. This search strategy identified other useful terms for local government, including 'city government'. On an initial scan of the literature and chosen key index terms, it was apparent that some language around HiAP extended to the underlying concepts, with terms used such as 'determinants of health' and 'health inequity' being a focus of the policy process, sometimes with and sometimes in the absence of the term HiAP. More general terms such as 'healthy public policy' returned largely irrelevant articles related mostly to health services or behavioural approaches to health policy action. Within Proquest, the full term of 'policy process' will be used instead of 'polic*' as this returned fewer though more relevant sources. A search on Informit database for 'health and all policies' and 'local government' was undertaken, returning nil results so this database was considered irrelevant for the search. Sources returned in Web of Science were limited and duplicated other databases, so was also considered irrelevant for the proposed search strategy.

A full list of final search terms is outlined in Table 1, with a summary of the search strings for each database and their results in Appendix I.

Table 1: Key words and search terms, using a PCC framework as a guide.

PCC	Key Terms	Alternative terms included
Context	Local government*	"Local council", Municipality, "city government"
Concept 1<br>**AND**	Health in all polic*	"determinants of health" or "health *equit*"
Concept 2	"polic*"	"policy process"

In addition to the database searches, a second strategy will be used to manually scan the included article references for further relevant sources that may not have been identified in the database searches.

Study/Source of Evidence selection

Following the search of databases, all identified citations will be uploaded into EndNote X9 and duplicates removed. The titles and abstracts from the final list of sources will be imported to Rayyan[1] software, in preparation for screening by two reviewers for assessment against the

[1] Mourad Ouzzani, Hossam Hammady, Zbys Fedorowicz, and Ahmed Elmagarmid. Rayyan — a web and mobile app for systematic reviews. Systematic Reviews (2016) 5:210, DOI: 10.1186/s13643-016-0384-4.

eligibility criteria of the review (Appendix II). Any disagreements for inclusion at this first stage will be discussed and main reasons for exclusion of sources noted.

All sources that are considered for further review will be retrieved in full text and assessed against the inclusion/exclusion criteria by two independent reviewers. The primary reason for exclusion of sources will be documented in a separate Microsoft Excel file and reported in the scoping review. Any disagreements on the inclusion of sources will be resolved by discussion in the first instance by the two reviewers or escalated to other reviewers if a decision cannot be reached. The results of the inclusion and exclusion process will be fully reported in the final scoping review.

Data Extraction

Data extraction will include source reference, level of government, country, concept focus, use of political science theory, methodology, key findings and relevance to the review questions (Appendix III). Any limitations or quality assessment of studies will be noted, though a formal quality appraisal will not be undertaken. A data extraction template is attached (Appendix II). The data extraction will be piloted by two reviewers using a sample of 3-4 sources. Any disagreements that arise will be discussed or escalated to other reviewers if required. Following this, the data extraction table will be refined if required. All further data extraction will be undertaken by one researcher and discussed with the second reviewer if there is any ambiguity.

Data Analysis and Presentation

The data will be presented in a diagrammatic and narrative form, including a bar chart representing the year sources were published, country in which the source was authored and use of political science theory within the research. A narrative account of the critical factors involved in the policy process will be themed into subheadings and discussed in relation to the evidence.

Acknowledgements

Courtney Moran, Liaison Librarian for the University of the Sunshine Coast, for her assistance with the search strategy.

Funding

No funding or possible conflicts of interest to disclose.

References

Breton, E., & De Leeuw, E. (2011). Theories of the policy process in health promotion research: a review. *Health Promotion International, 26*(1), 82-90. doi:https://doi.org/10.1093/heapro/daq051

Burris, S., Hancock, T., Lin, V., & Herzog, A. (2007). Emerging strategies for healthy urban governance. *Journal of Urban Health, 84*(1), 154-163. doi:https://doi.org/10.1007/s11524-007-9174-6

Collins, P., & Hayes, M. (2013). Examining the Capacities of Municipal Governments to Reduce Health Inequities: A Survey of Municipal Actors' Perceptions in Metro Vancouver. *Canadian Journal of Public Health. Revue Canadienne de Santé Publique, 104*(4), e304-310. doi:http://www.jstor.org/stable/canajpublheal.104.4.e304

Guglielmin, M., Muntaner, C., O'Campo, P., & Shankardass, K. (2017). A scoping review of the implementation of health in all policies at the local level. *Health Policy, 122*(3), 284-292. doi:https://doi.org/10.1016/j.healthpol.2017.12.005

Harris, E., & Wills, J. (1997). Developing healthy local communities at local government level: lessons from the past decade. *Australian and New Zealand Journal of Public Health, 21*(4), 403-412. doi:https://doi.org/10.1111/j.1467-842X.1997.tb01722.x

Koivusalo, M. (2010). The state of Health in All policies (HiAP) in the European Union: potential and pitfalls. *Journal of Epidemiology and Community Health, 64*(6), 500-503. doi:http://dx.doi.org/10.1136/jech.2009.102020

Marmot, M., Friel, S., Bell, R., Houweling, T. A., Taylor, S., & and Commission on the Social Determinants of Health. (2008). Closing the gap in a generation: health equity through action on the social determinants of health. *The Lancet, 372*(9650), 1661-1669. doi:https://doi.org/10.1016/S0140-6736(08)61690-6

Ollila, E., Baum, F., & Peña, S. (2013). Introduction to Health in All Policies and the analytical framework of the book. In K. Leppo, E. Ollila, S. Pena, M. Wismar, & S. Cook (Eds.), *Health in All Policies: Seizing opportunities, implementing policies* (pp. 3-24). Finland: Ministry of Social Affairs and Health.

Peters, M. D., Godfrey, C. M., Khalil, H., McInerney, P., Parker, D., & Soares, C. B. (2015). Guidance for conducting systematic scoping reviews. *International Journal of evidence-based healthcare, 13*(3), 141-146. doi:https://doi.org/10.1097/XEB.0000000000000050

Peters MDJ, G. C., McInerney P, Munn Z, Tricco AC, Khalil, H. (2020). Scoping Reviews. In M. Z. Aromataris E (Ed.), JBI Manual for Evidence Synthesis: JBI. doi:https://doi.org/10.46658/JBIMES-20-12

Shankardass, K., Renahy, E., Muntaner, C., & O'Campo, P. (2014). Strengthening the implementation of Health in All Policies: a methodology for realist explanatory case studies. *Health Policy and Planning, 30*(4), 462-473. doi:https://doi.org/10.1093/heapol/czu021

Shankardass, K., Solar, O., Murphy, K., Greaves, L., & O'Campo, P. (2012). A scoping review of intersectoral action for health equity involving governments. *International journal of public health, 57*(1), 25-33. doi:https://doi.org/10.1007/s00038-011-0302-4

Van Vliet-Brown, C. E., Shahram, S., & Oelke, N. D. (2017). Health in All Policies utilization by municipal governments: scoping review. *Health Promotion International*, dax008. doi:https://doi.org/10.1093/heapro/dax008

WHO and the Government of South Australia. (2010). The Adelaide Statement on Health in All Policies: moving towards a shared governance for health and well-being. *Health Promotion International, 25*(2), 258-260. doi:https://doi.org/10.1093/heapro/daq034

World Health Organization. (2012). *Addressing the social determinants of health: the urban dimension and the role of local government*. (9289002697). World Health Organization, Regional Office for Europe https://www.euro.who.int/en/publications/abstracts/addressing-the-social-determinants-of-health-the-urban-dimension-and-the-role-of-local-government

World Health Organization. (2014). *Health in all policies: framework for country action*. Helsinki, Finland https://apps.who.int/iris/bitstream/handle/10665/112636/9789241506908_eng.pdf?sequence=1

Appendices

Appendix I: Factors considered in the policy process

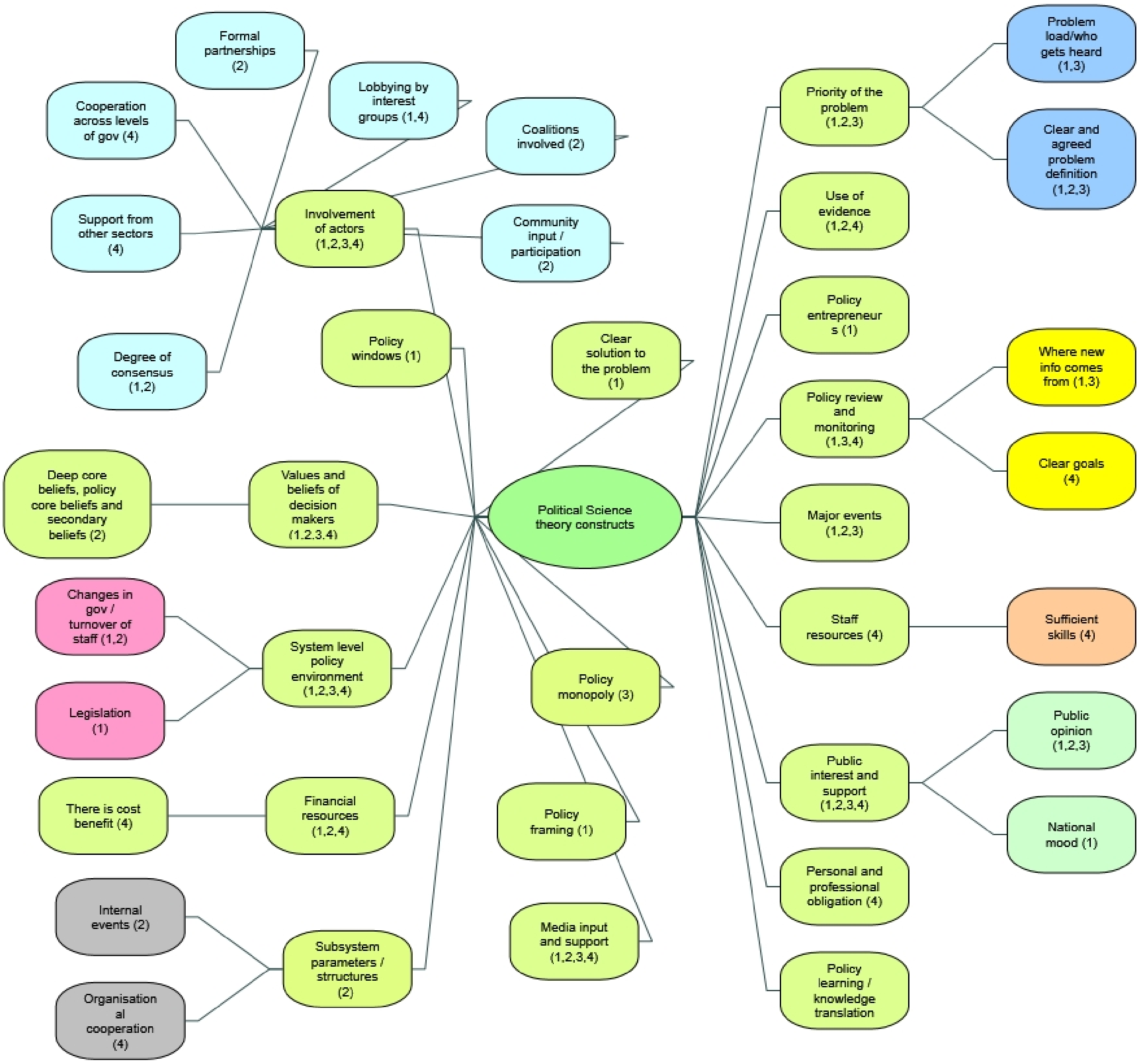

Figure 1: Factors considered in the policy process, based on the theoretical lens for the research (1=Multiple Streams Framework, 2=Advocacy Coalition Framework, 3=Punctuated Equilibrium Framework and 4=Analysis of Determinants of Policy Impact)

Appendix II: Search strategy

Database	Search string used	Limits	Number of sources
Proquest	("local government" OR "municipality" OR "local council" OR “city government”) AND ("health in all polic*" OR "determinants of health" OR "health inequit*" OR "health equit*") AND ("policy process")	**Year:** 1 Jan 2001-1 Jan 2021<br>**Language:** English<br>**Sources:** Dissertations & Theses, Scholarly Journals and Reports	593
Scopus	("local government" OR "municipality" OR "local council" OR “city government”) AND ("health in all polic*" OR "determinants of health" OR "health *equit*") AND ("polic*")	**Year:** 2001-12 March 2021<br>**Language:** English<br>**Sources:** Articles and Reviews	208
EBSCO	("local government" OR "municipality" OR "local council" OR “city government”) AND ("health in all polic*" OR "determinants of health" OR "health *equit*") AND ("polic*")	**Year:** 2001-Jan 2021<br>**Language**: English<br>**Sources:** Academic Journals, Journals, Dissertations	243

Appendix III: Exclusion and inclusion flow chart

Database settings: Restrict to limits: English, 2001-2020, exclude newspapers and conference abstracts

	Include	Exclude	Examples for exclusion
Is the study/case study focussed within the LG setting? (either solely or in conjunction with other tiers of gov)	Yes	No	Set in State or Federal tiers of government.<br>Set in local healthcare settings or community-based organisations. Not in any particular context.
Does the source refer to HiAP or a related concept e.g. health inequities?	Yes	No	Focus on biomedical or lifestyle factors e.g. obesity.
Does the source describe the policy process? (e.g. getting policy onto political agenda, factors enabling or challenging the development or implementation of policy)	Yes	No	Discusses content in policy documents or policy impacts/outcomes.

Appendix IV: Data extraction instrument

Study details and characteristics:	
Study citation:	
Year:	
Country:	
Level of government(s):	
Key concept of focus:	
Aim/objective of the research:	
Research methods:	
Research participants:	
Applied theory:	
Application of political science theory:	
Key findings - barriers and/or enablers identified in the policy process:	
Comparison of different context/jurisdictions:	
Study limitations:	
